# Soft Nanomembrane Sensor-Enabled Wearable Multimodal Sensing and Feedback System for Upper-Limb Sensory Impairment Assistance

**DOI:** 10.1021/acsnano.4c15530

**Published:** 2025-01-31

**Authors:** Tae Woog Kang, Yoon Jae Lee, Bruno Rigo, Ira Soltis, Jimin Lee, Hodam Kim, Gaorong Wang, Nathan Zavanelli, Eyas Ayesh, Wali Sohail, Houriyeh Majditehran, Scott H. Kozin, Frank L. Hammond, Woon-Hong Yeo

**Affiliations:** † Wearable Intelligent Systems and Healthcare Center (WISH Center), Institute for Matter and Systems, Georgia Institute of Technology, Atlanta, Georgia 30332, United States; ‡ George W. Woodruff School of Mechanical Engineering, Georgia Institute of Technology, Atlanta, Georgia 30332, United States; § School of Electrical and Computer Engineering, Georgia Institute of Technology, Atlanta, Georgia 30332, United States; ∥ Adaptive Robotic Manipulation Laboratory, George W. Woodruff School of Mechanical Engineering, Georgia Institute of Technology, Atlanta, Georgia 30332, United States; ⊥ Shriners Hospital for Children, Philadelphia, Pennsylvania 19140, United States; # Wallace H. Coulter Department of Biomedical Engineering, Georgia Institute of Technology and Emory University School of Medicine, Atlanta, Georgia 30332, United States; ¶ Parker H. Petit Institute for Bioengineering and Biosciences, Institute for Robotics and Intelligent Machines, Georgia Institute of Technology, Atlanta, Georgia 30332, United States

**Keywords:** nanomembrane, soft wearable, upper-limb sensory impairment, rehabilitation, tactile feedback, wireless electronics

## Abstract

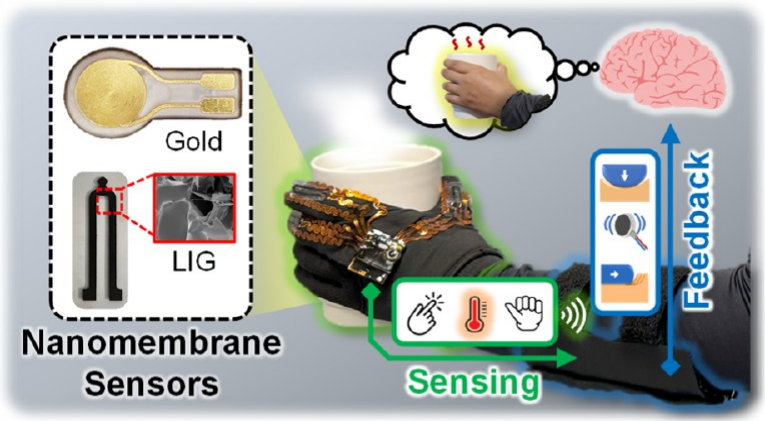

Sensory rehabilitation in pediatric patients with traumatic spinal cord injury is challenging due to the ongoing development of their nervous systems. However, these sensory problems often result in nonuse of the impaired limb, which disturbs impaired limb rehabilitation and leads to overuse of the contralateral limb and other physical or psychological issues that may persist. Here, we introduce a soft nanomembrane sensor-enabled wearable glove system that wirelessly delivers a haptic sensation from the hand with tactile feedback responses for sensory impairment assistance. The smart glove system uses gold nanomembranes, copper-elastomer composites, and laser-induced graphene for the sensitive detection of pressure, temperature, and strain changes. The nanomaterial sensors are integrated with low-profile tactile actuators and wireless flexible electronics to offer real-time sensory feedback. The wearable system’s thin-film sensors demonstrate 98% and 97% accuracy in detecting pressure and finger flexion, respectively, along with a detection coverage of real-life temperature changes as an effective rehabilitation tool. Collectively, the upper-limb sensory impairment assistance system embodies the latest in soft materials and wearable technology to incorporate soft sensors and miniaturized actuators and maximize its compatibility with human users, offering a promising solution for patient sensory rehabilitation.

Traumatic spinal cord injury (TSCI) is the damage to vertebrae or spinal cord due to the sudden traumatic impact on the spine,^1,2^ recognized globally as a major contributor to morbidity and mortality that significantly impairs the quality of life.^3,4^ TSCI may cause neuropathic pain,^5,6^ including motor or sensory deficits like hypoesthesia or paresthesia, which are sensory dysfunctions of body parts.^7,8^ In the pediatric ages, TSCI can result from various traumatic events, including shaken baby syndrome,^9^ sports injuries,^10^ falls, motor vehicle accidents, or violence.^11,12^ However, compared to adults, TSCI in the pediatric population presents unique challenges since their body systems are not yet fully developed, so they can be recovered properly.^4,13−15^ However, these sensory challenges often result in the rejection of the use of the affected limbs and unbalanced locomotion, further complicating rehabilitation. This potentially causes additional physical or psychological issues that may persist or even worsen over time during early childhood development. Current methods typically involve transplanting healthy nerves to areas affected by sensory loss, but they are often costly and invasive.^16,17^ Consequently, there is an urgent need for alternative strategies that are less invasive and better suited to the needs of pediatricians in sensory rehabilitation.

Wearable sensing gloves have a unique form-factor of assistive technology.^18,19^ These gloves, utilizing various sensing mechanisms,^20−24^ have found diverse applications in the recent years, from virtual reality^25,26^ to sign language detections^27^ and healthcare diagnostics.^28,29^ Recently, several researchers have explored the use of gloves to help patients with sensory impairments.^30^ Despite these advancements, challenges remain in making these sensory systems portable and easy to use during all-day rehabilitation.^31−33^ Additionally, the most reported rehabilitation systems are limited to transmitting a single type of sensory system.^34−36^ Moreover, currently developed gloves generally rely on rigid, bulky sensing components or circuits.^37−39^ Addressing these challenges requires innovation to make gloves more comfortable, lightweight, and portable, thereby improving their utility and practicality in actual life rehabilitation scenarios.^40^ Meanwhile, to effectively aid in rehabilitating patients with sensory impairments, it is crucial to transfer the detected sensory information to other parts of the body.^41,42^ Among various feedback systems, noninvasive systems such as vibrotactile,^43^ electrotactile,^44,45^ thermal,^46,47^ and mechanotactile, are preferred for their simplicity and convenience.^34,35,48^ However, current feedback systems typically support only single-sensory transmission,^36,49^ highlighting a growing need to develop systems that can deliver multimodal sensory inputs from the hand, encompassing not only pressure but other sensations as well.^48,50^ Recently, wearable and flexible electronics have emerged and expanded in various devices with nanomembrane structures with their intrinsic characteristics.^51−54^ These nanomembrane electronics have been developed with various materials, such as noble metals,^55^ conductive polymers,^56^ and carbon materials,^57^ featuring flexibility,^58^ ultrathin,^59^ stretchability, and lightweight properties.^59^ These characteristics are particularly promising for wearable sensing devices, offering high sensitivity, fast response times, and multifunctionality.^60,61^ These advantages have led to practical applications across a wide range of fields, including healthcare,^62^ the medical industry,^63^ smart homes,^64,65^ and various other fields of interest.^66,67^ Simultaneously, these promising applications require sensing electrodes that can capture a diverse range of parameters from the human body, including bioelectrical signals,^68^ strain,^63,69^ pressure,^70^ and temperature.^71,72^

Here, we introduce a soft nanomembrane-enabled, wearable, multimodal sensing feedback glove as a simultaneous haptic sensing and tactile feedback system designed to assist with daily hand rehabilitation in TSCI patients. This system externally delivers hand-sensory feedback to individuals, facilitating rehabilitation. The glove is integrated with multimodal sensors that detect multimodal hand sensory (pressure, temperature, and finger flexion) from the thumb, index finger, and middle finger, compensating for the sensory impairment patients.^73,74^ These sensors are fabricated with nanomembrane electronics those feature ultrathin, lightweight, and comfortable designs based on the recently developed wearable technologies,^59,75,76^ and with their characteristics and flexible electronics, the wearable sensor-integrated glove shows ultralight and compact design for pediatric hand rehabilitation.^40^ Then, the wearable multimodal tactile feedback system allows continuous collection and wireless transfer of haptic sensory data into tactile responses. This integrated sensor-actuator system’s performance is demonstrated in real-life scenarios with clearly identifiable system responses. With the promising wearable device and multimodal feedback capability, the multimodal sensing feedback glove provides potential direction for addressing sensory impairments and treating TSCI patients.

## Results and Discussion

**Figure 1:**
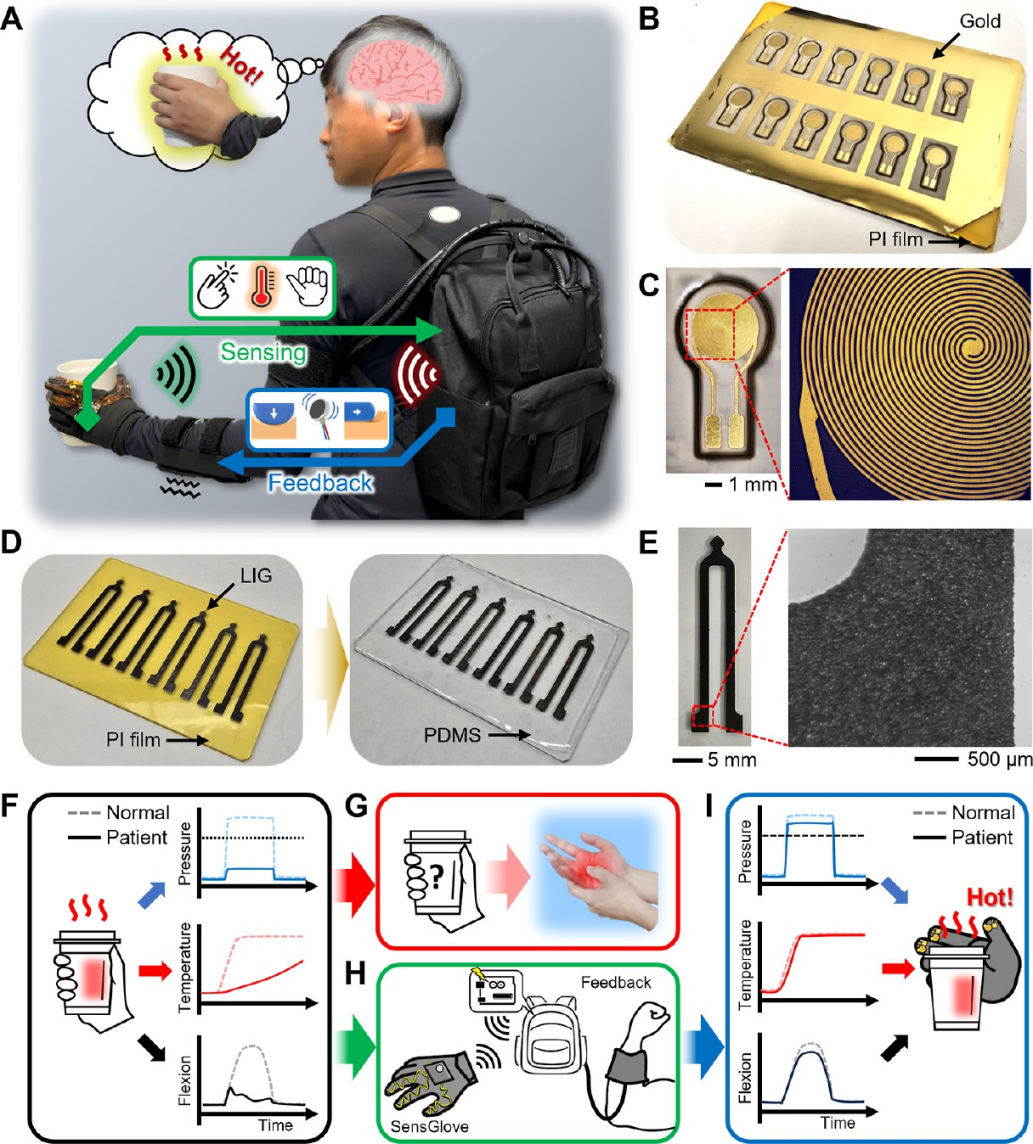
Overview of a soft wearable multimodal sensing glove and feedback system using various nanomaterials for upper-limb sensory impairment assistance. (A) Illustration of the integrated system, including wearable multimodal sensors in a glove and a band-shaped tactile feedback system. (B,C) Photos of fabricated nanomembrane temperature sensors in an array (B) and a single sensor (C). (D) LIG-based strain sensor for a wearable multimodal sensing glove. (E) Photo of a zoom-in view of the fabricated sensor. (F) Schematic illustration showing the difference in sensing capabilities between normal people and patients, possibly causing (G) unexpected injury risks to patients. (H) Schematic illustration of wireless communication between the soft wearable multimodal sensing glove and the feedback system. (I) Illustration showing the wearable system’s capability in detecting pressure, temperature, and flexion using nanomembrane sensors.

This article introduces a wearable multimodal sensing feedback glove (Figure 1A), addressing the rehabilitation challenges faced by TSCI patients with sensory impairments. The wearable multimodal sensing feedback glove was designed with miniaturized flexible and ultrathin sensors that were comfortable and lightweight. The temperature sensor uses ultrathin gold (Au) membranes, and its spiral structure induces resistance to help monitor temperature changes (Figure 1B,C). The strain sensor for finger flexion uses a polyimide (PI) film. An ultraviolet (UV) laser patterns the PI film, generating high-quality laser-induced graphene (LIG) (Figure 1D,E). This method offers high reproducibility and high mass production capabilities. Pediatric TSCI patients often exhibit reduced pressure sensitivity,^77,78^ weak or delayed temperature responses,^79,80^ and limited finger flexion responses against practical input compared to individuals without sensory impairment (Figure 1F).^77,81−83^ These sensory limitations hinder their ability to perceive and react to the physical and chemical features of the objects they are holding or interacting with, increasing the risk of burns and injuries (Figure 1G).^84,85^ However, the currently available sensory assistance gloves still use heavy (over 100 g), rigid, and bulky sensors and circuits that give unnecessary weight to the hand.^37−39^ This is particularly problematic for pediatric patients, who are smaller and less physically strong. Also, they have only single sensory feedback transmission, mostly pressure sensing, which is insufficient for multimodal sensory transmission from the hand (Table S1). An ultralight, wearable multimodal sensing feedback glove has been developed to address these issues. When a patient grips a hot object, the wearable sensing feedback glove detects changes in pressure, temperature, and finger flexion, processes this information in real time, and transfers it back to the individual (Figure 1H). Consequently, pediatric TSCI patients can recognize object features almost as if they had normal sensory responses, enabling quick and appropriate reactions. This process involves pressure responses, rapid detection of temperature changes, and swift finger flexion responses, so the sensing feedback glove can significantly reduce the risk of burns and injuries in pediatric TSCI patients. Furthermore, the wearable multimodal sensing feedback glove helps align the patient’s sensory responses with those of a healthy individual through fast sensory transmission (Figure 1I). This enables pediatric TSCI patients to instantly identify potentially harmful conditions or other features of objects and respond swiftly, thereby enhancing both rehabilitation safety and effectiveness in daily interactions with objects.

**Figure 2:**
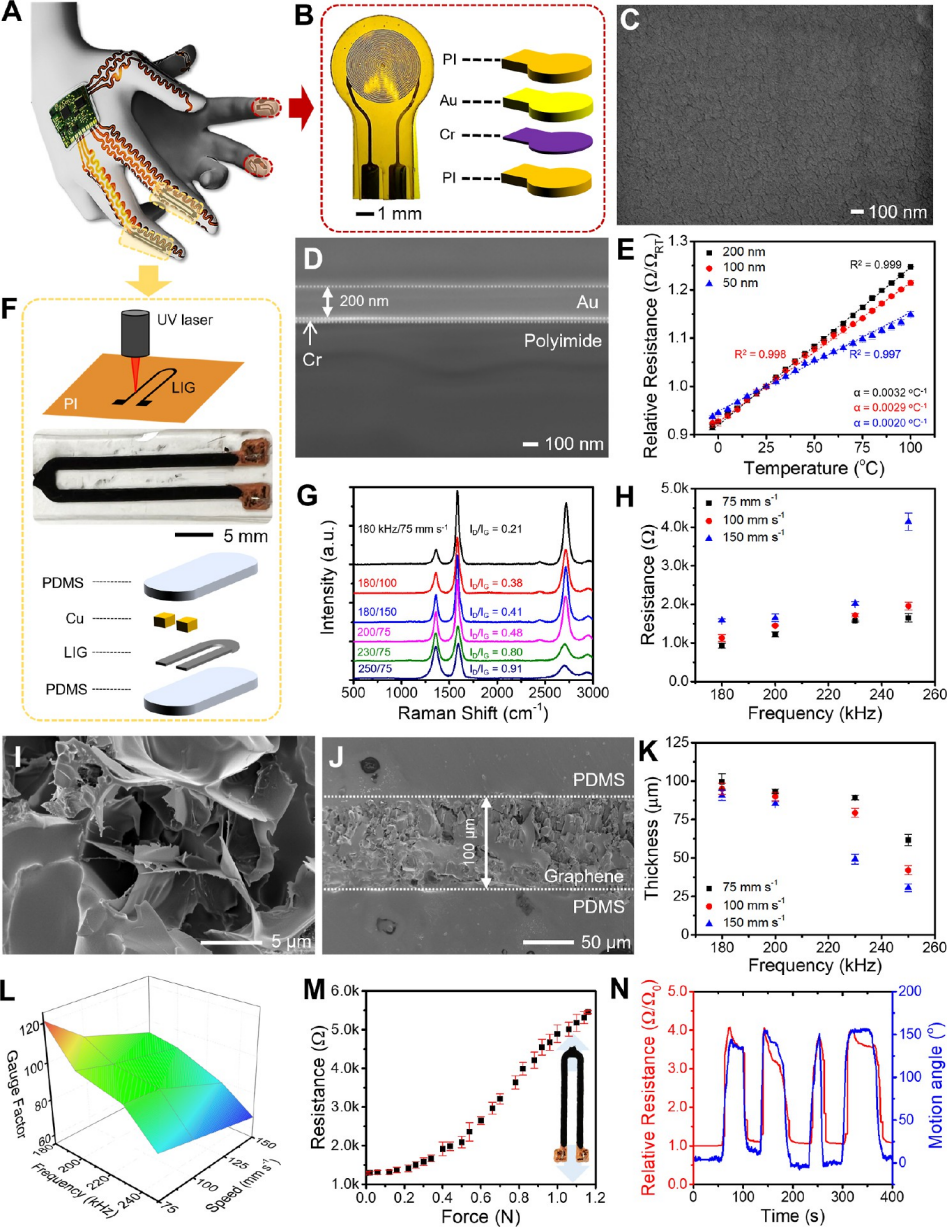
Design of the wearable multimodal sensing glove for haptic sensation. (A) Illustration of a wearable system integrating nanomembrane pressure, temperature, and strain sensors. (B) Detailed structure of a resistive temperature sensor. (C) Scanning electron microscopy (SEM) shows the top-view image and (D) cross-sectional image of the 200 nm-thick Au membrane on polyimide. (E) Relative resistance of multiple temperature sensors according to temperature changes. (F) Detailed structure of a LIG-based strain sensor. (G) Raman spectra and graphene D/G peak ratio of the strain sensor with different laser frequencies and scan rates. (H) Resistance changes of the strain sensor with different laser frequencies and scan rates. (I) Top-view image and (J) cross-sectional image of the LIG sensor with 180 kHz and 75 mm s^–1^ preparation condition. (K) Thickness difference of LIG on the strain sensors. (L) 3D mapping image for gauge factors of strain sensors with different manufacturing conditions. (M) Resistance response of the strain sensor according to forces. (N) Relative resistance changes of the strain sensor, while bending a finger.

Figure 2A illustrates the nanomembrane sensor-integrated wearable multimodal sensing glove. These sensors were strategically placed on the thumb, index finger, and middle finger—the primary digits involved in most tasks—and are connected to a flexible printed circuit board (fPCB) via thin serpentine interconnectors. The flexible temperature sensor was fabricated with a microscale spiral-patterned Au disc (Figure S1). Ultrathin Au was deposited on the PI film by using an e-beam metal evaporation method. To optimize the thickness of Au for the temperature sensor, it was deposited with different thicknesses of 50, 100, and 200 nm. SEM images (Figures 2B,C, and S2) revealed that the 50 nm-thick Au plate exhibited a rough surface with small grains. As the thickness increased, the surface became smoother, with larger grains (Figure 2D). Ultrathin Au plates were then laser-scribed with a femtosecond laser micromachine to make a spiral structure patterned as 40 μm width and 25 μm spaces. Then, the Au disc was covered with another PI film with remaining connection spaces, and silver chloride paste was used to connect the temperature sensor and copper connector for measuring resistance. The resulting sensors showed 4467 ± 2.8 Ω for 50 nm thickness, 1141 ± 4.9 Ω for 100 nm, and 687.3 ± 2.3 Ω for 200 nm at room temperature (Figure S3), which are much higher than those for pristine gold (0.3 Ω) due to the enhanced length by the spiral patterning. The temperature sensor followed linear dependency as a traditional conductive material formula with the following
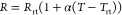
1where *R* is resistance at the temperature *T*, *R*_rt_ is resistance at the room temperature (*T*_rt_), and α is the temperature coefficient of resistance (TCR) for the material.^86^ This equation can be shifted to the correlation between the relative resistance and the temperature.
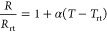
2

In Figure 2E, the TCR decreases according to reduced thickness, indicating a linear dependency of the relative resistance with a temperature change from 0 to 100 °C.^87,88^ The measured TCR of sensors are 0.0032, 0.0029, and 0.0020 °C^–1^ for 200, 100, and 50 nm, respectively. The decrease of the coefficient could be observed from the nanothickness metal electrodes, which was caused by the limited electron movement.^89^ The temperature sensor was optimized with a thickness of 200 nm due to its measurable resistance and high TCR. Also, it showed extraordinary linear responses against a wide temperature range from −3 to 100 °C with *R*-squared = 0.999, which is an exceptional property compared with the previously reported wearable temperature sensors (Table S2). The strain sensor for the flexion motion was designed with LIG.^90^ The LIG strain sensors integrate multiple layers to form a flexible configuration (Figures 2F and S4). Initially, when irradiated with a laser at 150 kHz frequency and 75 mm s^–1^ scan speed, the resulting LIG had a rigid structure unsuitable for strain sensing due to its low quality.^91^ Therefore, we optimized the LIG fabrication by adjusting laser frequencies between 180 and 250 kHz and scan speeds from 75 to 150 mm s^–1^. The fabricated LIGs show Raman scattered peaks under 485 nm laser at 1360, 1582, and 2720 cm^–1^, corresponding to the graphene’s D, G, and 2D peaks (Figures 2G and S5).^92^ The LIG produced at 180 kHz and 75 mm s^–1^ showed sharp peaks with a D/G peak ratio of 0.21, indicating a low defect density in the graphene structure. The peak ratio was increased as the scan speed or frequency increased (Figure S6). The DG peak ratio was increased to 0.91 at 200 kHz and 75 mm s^–1^ scan speed, and it was shifted to about 1 with an increasing scan speed because the low energy of the laser does not fully graphitize them. Resistance of the LIGs shows DG peak ratio-dependent properties from 295.0 ± 4.6 Ω to 2057.7 ± 11.7 Ω (Figure S7). The resistance value increases from 932.7 ± 60.0 Ω to 4143.7 ± 224.4 Ω (Figure 2H) after being transferred to the PDMS due to defect generation. LIGs show a 3-dimensional porous multigrain structure and could not be observed significantly (Figures 2I and S8).^93^ However, vertical section SEM shows different thicknesses with varying laser conditions (Figures 2J,K, and S9). A lower frequency of the laser offers a higher energy to fuse and graphitize deeper PI powders to form a thicker graphene.^94^ The gauge factor of each LIG was then measured to optimize the fabrication conditions within a strain range of 0% to 20%, which corresponds to the typical range of finger flexion. The gauge factor is measured by the following equation
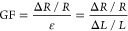
3where ε is the applied strain, which is equal to the change of length (Δ*L*) from the original length (*L*), and Δ*R* is the change of resistance from the original resistance (*R*) of the LIG while a strain is applied. While the strain force was applied to induce the length of LIGs from 2.5 to 3 cm, the resistance of LIGs gradually increased (Figures 2L and S10). LIG was optimized with the laser condition of 180 kHz and 75 mm s^–1^, which showed the highest gauge factor of 121 among all fabricated sensors, which could be derived from a low defect ratio, making LIG sensitive to generated defects. It showed higher GF than the previously reported strain sensors under mild strain conditions (Table S3). Copper was reductively deposited on the tails of LIG to connect the interconnector with LIG. The LIG strain sensor was then tested with an elongation, and it showed an elongation-force-dependent resistance increment (Figure 2M). To validate the LIG strain sensor for finger flexion detection, we analyzed the resistance changes corresponding to bending motions. The strain sensor was securely positioned on the finger section of a glove, and its resistance was compared with the angle of finger flexion. The total bending angle was determined as a motion angle, and it was calculated from the total angle changes from each hinge. As the finger was bent, the motion angle was increased to about 150°, and the resistance was consequently increased to four times larger than at the stage of 0°. Figure 2N presents the results of four bending and releasing cycles over 400 s, with each state maintained for a set duration. The resistance changes closely corresponded to the finger flexion, demonstrating the sensor’s reliability. For pressure sensing, flexible PDMS-based dielectric capacitors were fabricated using a simple method (Figure S11).^95^ Both sides of the 0.35 × 0.35 cm flexible copper foil (300 μm thickness) layers were fabricated by a femtosecond laser micromachine and then covered with PI by a thermal press, and they are closely fixed by the 80 μm of PDMS layer (Figures S12 and S13). The capacitance of the sensor follows the equation
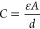
4where *C* is capacitance in Farads, ε is the absolute dielectric permittivity of the material, *A* is the area of layer overlap in square meters, and *d* is the distance between layers in meters.^96^ Absolute dielectric permittivity can be derived as
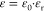
5where ε_0_ is the electric constant (8.854 × 10^–12^ F m^–1^) and ε_r_ is the relative permittivity of the dielectric materials. The pristine pressure sensor shows 3.65 pF, and the experimentally calculated ε_r_ was 2.692, which is the same parameter as the previously reported ε_r_ of the bulk PDMS.^97^ Then, the fabricated PDMS-based capacitance sensors measured their pressure response against applying force from 0 to 95 N using a force and torque measurement instrument, which is an acceptable measurement range for finger force.^98^Figure S14a shows a change in the capacitance of the sensor with different forces. When force is applied, the distance *d* of the dielectric layers is reduced with the compressed PDMS layer.^99^ The capacitance of the pressure sensor is increased as following the eq 4.^100^ The capacitance is decreased by decompressed PDMS after the force is released. The capacity change of the PDMS-based capacitor showed a hysteresis graph because of its viscoelastic properties (Figure S14b).^101^

**Figure 3:**
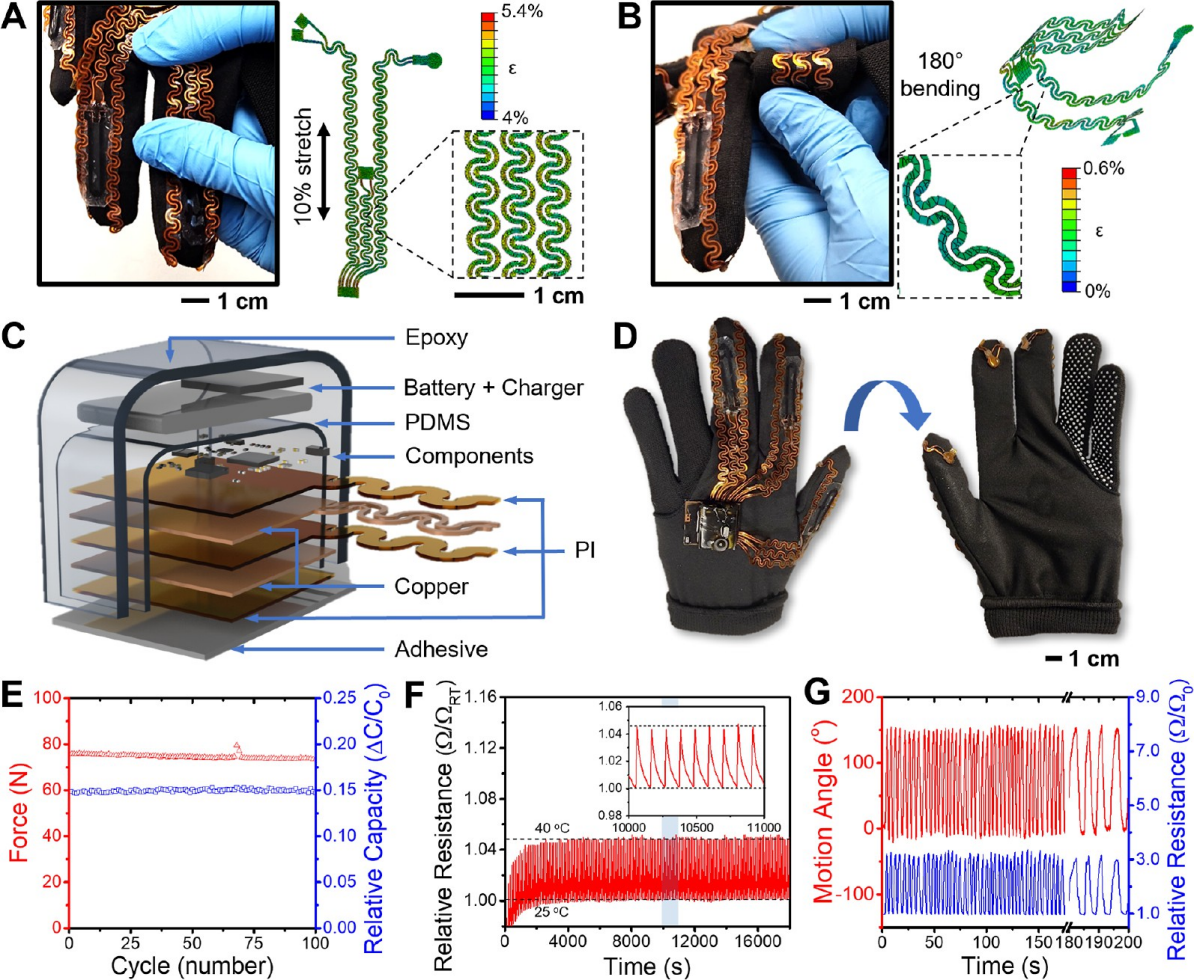
Mechanical characterization of the wearable multimodal-sensing glove. (A,B) Comparison of experimental study and computational modeling of stretchable interconnectors upon applied tensile strain 10% (A) and upon 180° bending (B). (C) Illustration showing a wireless integrated circuit using multilayered structures and electronics components. (D) Photos showing a fabricated glove with the integrated electronics on the front side of the glove, while the backside shows membrane sensors. (E) Long-term stability of the pressure sensor’s performance upon applied pressure changes. (F) Long-term stability of the micro spiral temperature sensor under temperature changes between 25 and 40 °C. (G) Comparison of the wearable multimodal sensing glove’s finger flexion bending and strain sensor resistance change.

To connect sensors and fPCB, the serpentine-structured interconnector has been fabricated to give strength against elongation caused by finger bending (Figures S15 and S16).^25,102^Figure 3 summarizes the mechanical characteristics of the stretchable interconnectors and integrated circuits. Finite elemental analysis was used to optimize the serpentine structure.^65^ The stretchable interconnectors show low tensile stress against 10% stretch and 180° bending test, which are reasonable ranges by finger flexion (Figure 3A,B). The interconnect showed a maximum strain of 16.75% against elongation. fPCB was designed for wireless Bluetooth connection to transfer multimodal sensory data and integrated with a rechargeable battery (Figures 3C and S17). The wearable multimodal sensing glove was finally manufactured for haptic sensation with the integration of the sensors and the fPCB circuit onto the glove via PDMS (Figure 3D). The temperature and pressure sensors were placed on the distal phalanx of the thumb, index finger, and middle finger, and the finger flexion sensors were placed on the proximal interphalangeal joints to collect hand sensation and deliver to the feedback system effectively,^103^ and they were fixed on the glove with PDMS. The little finger and ring finger area were placed on nonslip pads to reduce slip while gripping an object. To validate the performance of the wearable multimodal sensing glove, it was tested for the cyclability of integrated pressure sensors under repeating force. The pressure was applied by repeating the hammer for the same distance. The glove showed less than a 2% reduction in capacitance in response to the applied force, even after 100 repetitive applications, as shown in Figure 3E. The temperature sensors of the glove were tested under repeated temperatures from 25 to 40 °C by a thermogenerator. The resistance of the temperature sensor increased as the finger’s temperature reached that of the thermogenerator. As shown in Figure 3F, the sensor shows reliable resistance change against the temperature changes, with a fast response delay under the temperature change from 40 to 25 °C, about 0.24 ± 0.6 s (Figure S18). The glove tested how pressure affects the temperature sensor and how temperature affects the pressure sensor. The results showed that each sensor exhibited negligible changes when exposed to the other’s stimulus, confirming that they do not interfere with each other, even when placed close together (Figures S19 and S20). The strain sensor was also validated by repeating the finger flexion (Figure 3G). The index finger was used for the measurement of strain change, and the finger was repeatedly bent about every 5 s. The strain sensor’s resistance variation is closely correlated with the changes in the motion angle, demonstrating a high concordance rate of 96.6%. The results showed that the wearable multimodal sensing glove featured high multimodality (pressure, temperature, and flexion) as well as good flexibility from the flexible sensor, interconnector, and fPCB compared to other sensing gloves (Table S4). Also, it adds only 18.77 ± 0.58 g to the hand, making it ultralight compared to the other wearable sensing gloves.^37,39,40,104,105^ Additionally, since the wearable multimodal sensing glove was designed with soft nanomembrane fabrication, the cost was estimated to be only $ 13.4, including a commercial glove, circuits, and battery due to the small amount of material used.^106^ This cheap, compact, and almost negligible weight multimodal sensing glove helps with daily wear for pediatric TSCI without causing discomfort or fatigue. The proposed wearable multimodal sensing glove would not hinder the daily movement of the hand.

**Figure 4:**
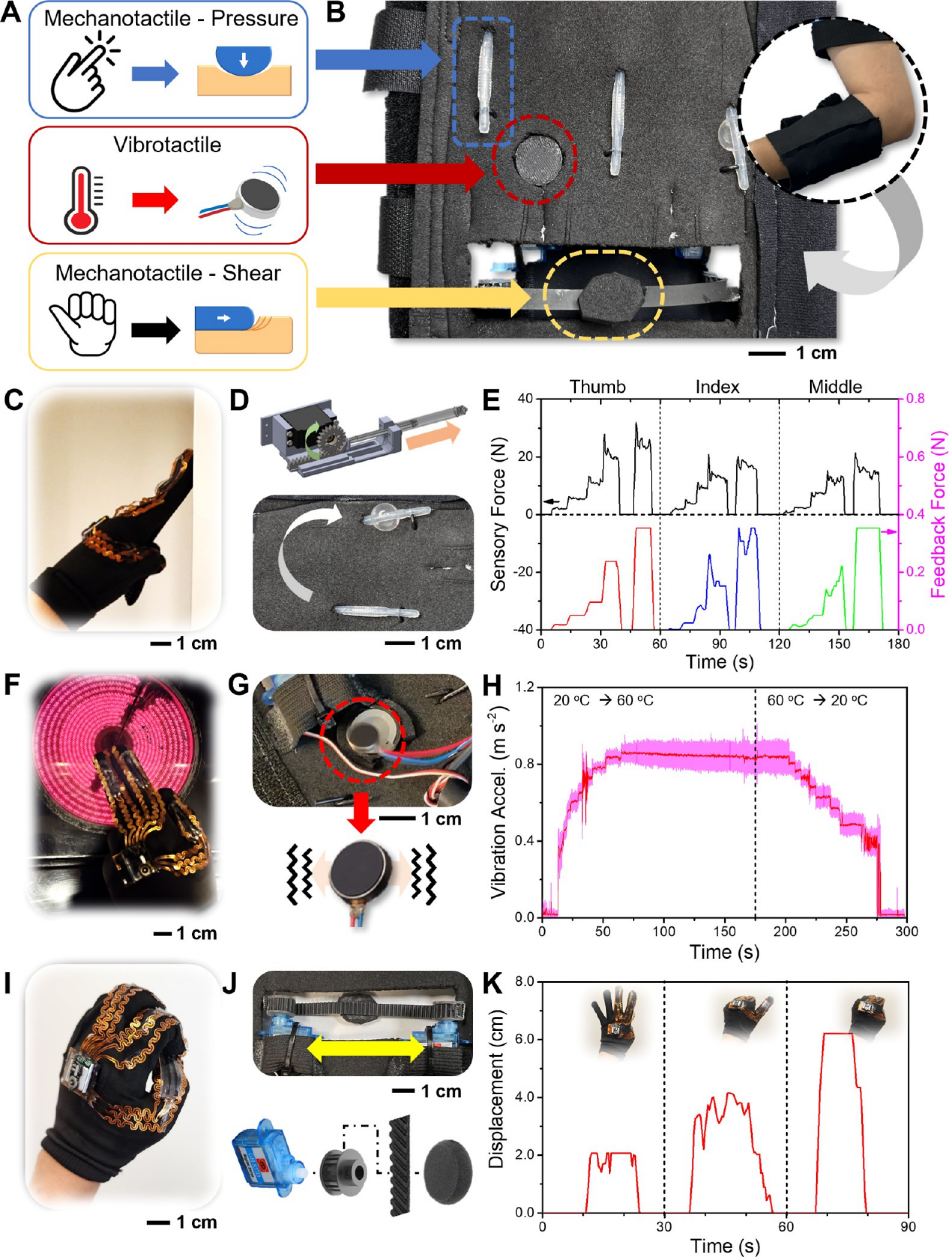
Design and characterization of the tactile feedback system. (A) Description of the converting type of feedback responses from each signal of the wearable sensing glove. (B) Velcro band with integrated actuators. (C) Photo showing an activity to push the wall with the glove. (D) Operation mechanism of the mechanotactile pressure actuator. (E) Comparison between sensory force input and responded feedback force. (F) Photo showing an activity to touch a hot surface with the glove. (G) Operation mechanism of the vibrotactile temperature actuator. (H) Vibrotactile response according to the change of the glove temperature from 20 to 60 °C, and 60 to 20 °C. (I) Photo showing an activity to bend fingers. (J) Operation mechanism of the responded displacement actuator. (K) Displacement change of the finger flexion feedback while moving the hand with different gestures.

The feedback system was designed as an external wireless delivery system from the glove to apply pediatric TSCI rehabilitation safely.^107^ It also reduced weight loading on the pediatric arm, which helped balance pediatric development.^108−110^ The feedback delivered sensory information to individuals immediately with negligible communication delay (up to 10 ms), and a tactile feedback system converted each sensory input into a different response method with a fast elapsed time (Figure S21). The feedback system consisted of a control box that contained a battery, an Arduino microcontroller, mechanical and electrical components (Figure S22), and a Velcro band to deliver feedback responses to the arm directly. Mechanotactile pressure, linear displacement, and vibrotactile methods were chosen for the pediatric rehabilitation feedback system since they are less discomfort and deliver signals safely.^18,43^ The measured pressure change from the glove was converted with mechanotactile pressures by the diameter change of balloons, and the change of the strain by finger flexion was converted with a linear displacement response. The temperature signal was transferred to the vibration frequency of the vibrotactile system. To make the feedback system in the limited area of the Velcro band compact, the temperature and strain changes from the fingers were delivered as one response system. Figure 4 shows a wearable integrated actuator system delivering tactile feedback to the user. As shown in Figure 4A,B, the inside of the Velcro band had three balloon tubes for the pressure responses, a vibrate generator for the response of maximum temperature of fingers, and a displacement motor to reflect the whole finger flexion status. To validate the Velcro band-shaped feedback system of the wearable multimodal sensing glove, response signals were measured when pressure, temperature, and finger strain changes arose from the wearable multimodal sensing glove. Figures 4C–I describe the operation of the glove to measure each feedback response. For the pressure feedback system, pneumatic syringes in the portable bag gave pressure changes to the balloons of the Velcro band (Figure 4D and Supporting Information Video S1). The pressure response is first determined by the pneumatic rotor degree. When the rotor was rotated from 0° to 180°, the size of the balloon expanded so that the output pressure increased (Figures S23 and S24). After the characterization of the balloon changes by a pneumatic syringe, the pressure responses from each finger were tested with the force and torque measurement instrument. As shown in Figures 4E and S25, each feedback response from different fingers was well matched with directly measured sensory force. For the temperature feedback system, vibrotactile was given varying the intensity input from 0 to 255 based on the pulse-width modulation (PWM). The vibrotactile output could be measured by vibration sound frequency and acceleration (Figure S26).^111^ When the input intensity was higher than 80, the vibration could be measured, so the vibrotactile was used with an input intensity range of 80 to 255. The range was derived with 20 intervals (from 80 to 240, a 15 interval for 255) and was matched to the temperature range from 20 to 65 °C with 5 °C intervals (Figure S27). The vibration acceleration clearly demonstrated the temperature changes (Figure S28). The glove was then placed on the hot plate to change the temperature of the glove. When the glove was placed on the hot plate (60 °C), the vibration acceleration rapidly increased due to the frequent motion (Figure 4H). When the glove was placed on the cool mat (20 °C), vibration acceleration gradually decreased, and it was turned off after the temperature reached 20 °C. Also, the vibration changes could be monitored in its sound tones since high frequency generates a high tone (Supporting Information Video S2). Strain change of the glove was delivered with a linear displacement response for simplified notification (Figure 4J).^112^ The maximum displacement response was regarded as the whole finger being bent. Figure 4K shows the change of the displacement response with different finger motions. Bending the thumb resulted in a feedback response of only one-third of the maximum displacement, whereas bending both the index and middle finger to simulate a “thumbs up” gesture allowed for a feedback response of up to two-thirds of the total displacement. Maximum displacement could occur when all the sensors are bent by hand gestures such as “rock” (Supporting Information Video S3). The wearable multimodal sensing feedback glove, an integrated platform of the wearable multimodal sensing glove and tactile feedback system, was subsequently tested on a participant to evaluate its efficacy in aiding hand sensory rehabilitation.

**Figure 5:**
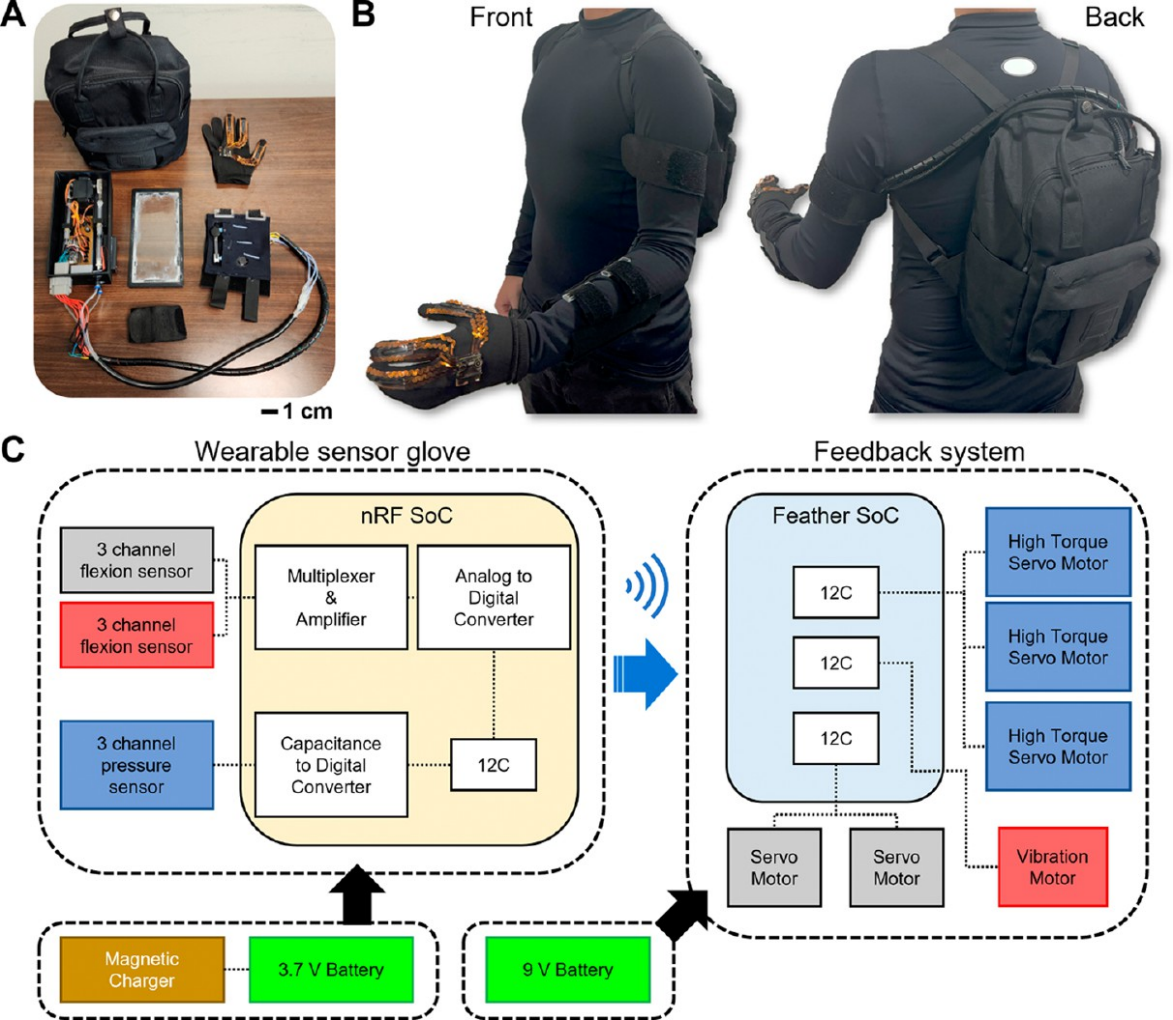
Integrated wearable multimodal sensing and feedback system. (A) Photo of the wearable system, including a multimodal sensing glove, an actuator suite, and a backpack. (B) Front and back images of the wearable multimodal sensing feedback system worn by a user with a glove on the left-hand. (C) Flowchart showing the overview of the system components, data acquisition by multiple sensors, wireless data transmission to a feedback system, and corresponding actuation of the feedback components using motors.

Figure 5A illustrates the individual components of the system prior to being worn. The feedback controller, which includes an Arduino microcontroller and a portable power source, is compact enough to fit into a small backpack, as shown in the front and back images of the system in use (Figure 5B). The wire connecting the armband to the feedback controller is securely fastened along the arm for safety and convenience. Figure 5C describes the block diagram of the system, detailing the integration of the sensing glove with the feedback system. The glove serves as a central hub for various three-channel sensors, including bend, temperature, and pressure sensors. Specifically, it interfaces with a three-channel bend sensor, a three-channel temperature sensor, and a three-channel pressure sensor. The nRF System on Chip (SoC) was designed with a multiplexer and amplifier and an analog to an analog-to-digital converter to process and relay the information from these sensors. Additionally, it computes a capacitance-to-digital converter for accurate real-time force measurement. The glove is powered by a 3.7 V LiPo battery and is expected to be used for 30 h with a 70 mA h battery, which is rechargeable via a magnetic charging interface.

**Figure 6:**
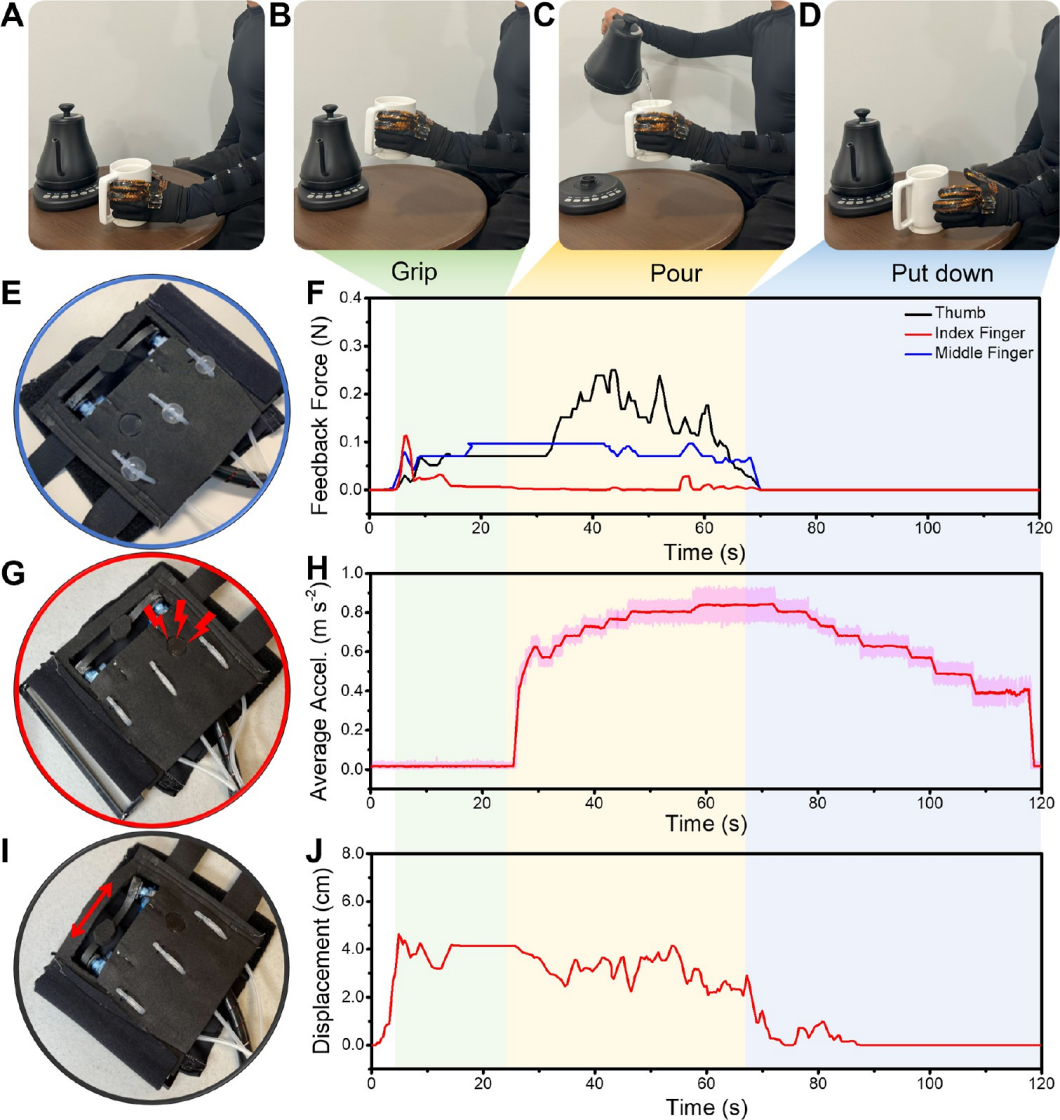
Demonstration of a real-world application of the wearable multimodal sensing and feedback system. (A–D) Photos showing an example where a subject wearing the multimodal sensing feedback glove reaches out to a mug, (B) gripping the mug, (C) pouring hot water, and (D) putting down the mug on the table. (E) Photo of the mechanotactile pressure feedback responses and (F) measured feedback forces during the sequential actions (A–D). (G) Photo of the vibrotactile temperature feedback response and (H) vibration acceleration changes due to a hot mug gripping. (I) Photo of the displacement response from the finger flexion and (J) measured displacement feedback.

To demonstrate the practical application of the wearable multimodal sensing feedback glove, an actual life scenario involving hot water pouring into a mug has been tested. This scenario is broken down into distinct stages, as shown in Figure 6: the pregrip phase (“before gripping a mug”—Figure 6A), the action of gripping and lifting the mug (Figure 6B), the process of pouring hot water while maintaining grip (Figure 6C), and finally, setting the mug down postpour (Figure 6D). The effectiveness of each component of the tactile feedback system was evaluated across these stages. During the gripping phase, the participant first contacted the mug with their index and middle fingers, followed by the thumb to secure the grip with different forces.^103,113^ After hot water was poured into the mug, the pressure of the thumb increased and became unstable because the mug got heavier (Figure 6E,F). The pressure feedback responses returned to the initial state after putting down the mug. The vibrotactile response remained constant during the gripping phase. When hot water was poured, the vibrator started to make vibrations immediately, and the vibration acceleration and sound frequency were increased until the subject put down the mug (Figure 6G,H). After the mixture was put down, the vibration response gradually decreased and returned to its baseline state. The strain response seemed similar to the pressure response. Similar to the pressure feedback, the strain response appeared to correlate with the gripping and pouring actions, as indicated in Figure 6I,J. The strain was altered exclusively during these specific actions. The experiment was repeated with the armband worn in reverse to demonstrate the functionality of the feedback response system (Supporting Information Video S4). The wearable multimodal sensing feedback glove was also demonstrated in different scenarios. Finger flexion could be clearly changed when a ball of different size was gripped (Supporting Information Video S5 and Figure S29). The vibrotactile response was also clearly demonstrated with different temperatures of the beaker. The wearable multimodal sensing feedback also tested significantly endangered situations for pediatric TSCI patients against high pressure or temperature. The glove quickly detects changes in these conditions, such as during door jamming or thermal contact, and alerts the patient to potential danger (Figures S30 and S31). The wearable multimodal sensing feedback glove showed exceptional performance in this complex test, suggesting its applicability for various daily activities, from simple to more intricate motions.

## Conclusions

This paper reports on a wearable, multimodal nanomembrane sensor-integrated glove, and a tactile feedback system to assist in rehabilitation for the upper-limb sensory impairments of TSCI patients. By integrating nanomaterials, soft sensors, flexible electronics, and low-profile actuators with a glove, we have developed a system that is comfortable for the wearer and highly effective in delivering multimodal sensory feedback. The enhanced material properties of the packaged sensors and actuators ensure effectiveness in delivering multisensory information under severe repetition cycles, 98% accuracy for the pressure sensor after 100 continuous cycles, comprehensive temperature coverage, and a 96.6% consistency in finger flexion. With enhanced reliability and connectivity, the tactile system transforms sensory inputs into immediate augmented responses essential for real-time rehabilitation exercises. The wearable multimodal sensing feedback glove demonstrates its effectiveness in supporting various daily activities, making it a highly effective tool for enhancing upper-limb rehabilitation. Moreover, the wearable multimodal sensing feedback glove can be adapted for other patients with sensory impairments requiring hand rehabilitation. The system’s robust performance and precise sensory feedback highlight its potential as a significant advancement in wearable rehabilitation technology.

## Materials and Methods

### Materials

Copper foil (6 μm, MSE Supplies), polyimide tape (Kapton tape, Bertech), polyimide film (Kapton film, Dupont), polydimethylsiloxane (PDMS), copper sulfate hexahydrate (CuSO_4_, Sigma-Aldrich), silver chloride epoxy (MG chemicals), solder paste (Chip Quik), and tacky flux (Chip Quik) were purchased for manufacturing and soldering the wearable sensing glove. 3 mL syringe (LabAider), motor (MG996R, Tower Pro), pneumatic push connector (ROZESAZZ), catheter tube (United States Plastic Corp.), Foley catheter (Medline Industry), submicro motor (SG51R, Tower Pro), vibration motor (Adafruit), and Adafruit Feather nRF52840 Express (Adafruit) were purchased for manufacturing the feedback system.

### Instruments

A femtosecond infrared laser micromachine (OPTEC) was used to microfabricate the pressure sensor, temperature sensor, and interconnector. A force measurement instrument (Mark-10) was used to measure the capacity of the pressure sensor, strain resistance of the finger flexion sensor, and the interconnector. An e-beam evaporator (Denton Explorer) was used to deposit gold (Au). A hot press (Rositek) was used for thermal attachment between polyimide films. An infrared thermal imaging camera (FLIR) was used to monitor the temperature and characterize the temperature sensor. SEM (scanning electron microscope, Hitachi) was used to characterize the ultrathin Au plate and LIG. Raman spectroscopy (Renishaw) was used to characterize the LIG. A UV laser platform was used to prepare the LIG. The CO_2_ laser was used for laser cutting of the 3D-printed structures, and a 3D printer (Stratasys; MakerBot) was used for manufacturing the feedback system supporting structures.

### Sensor Fabrication

For the microscale spiral temperature sensor (Figure S1), PDMS was spin-coated on the glass slide. The PI film was then covered on the glass slide, and Au was deposited. To make a stable PI/Au layer, 10 nm of Cr was deposited by e-beam evaporation under 1 Å s^–1^, and 50, 100, and 200 nm of Au was then deposited with a condition of 5 Å s^–1^. After the ultrathin Au plate was fabricated, a femtosecond IR laser was scribed for the spiral pattern. The pattern revealed about 40 μm of width with 25 μm space, and excess Au/PI film was peeled off. To make a PI cover for the Au pattern, another PI film was placed on the PDMS and cut by a femtosecond laser with an empty space for connection to the interconnector. The hot press at 400 K fixed the PI cover on the Au pattern for 30 min. The AgCl/epoxy composite was used to connect with the interconnector. AgCl/epoxy was taking place between the temperature sensor and the interconnector; it was hardened under 60 °C. A hot plate was used to measure resistance changes under different temperatures, and each temperature was confirmed by an FLIR thermal camera. The finger flexion strain sensor was designed using LIG (Figure S4). The PI film was placed on the glass slide, and a UV laser (355 nm, 30 W with 24%) was scribed on the PI tape to generate graphene. The surface of LIG was spin-coated by PDMS, and PDMS was cured to ensure that LIG was transferred to the PDMS. After peeling off the LIG from the PI film, LIG was then dipped in the CuSO_4_ solution, and Cu was reductively deposited on the edge of the LIG. After the Cu was soldered with Cu wire, the other side was also covered by the PDMS. Finally, the prepared strain sensor was soldered with the interconnector. The resistance change of the sensor against the strain was measured by Mark-10, and the comparison of the finger bending took place while the finger was bent. Each finger hinge was automatically captured from the video, and the summation of the total flexion angle was defined as the motion angle. The motion angle was compared to the resistance change of the strain sensor, which was measured using a multimeter. The pressure capacitive sensor was prepared with thin copper plates with a PDMS dielectric layer (Figure S12). A 300 μm of Cu foil was placed on the PDMS, and a femtosecond infrared laser (1030 nm) was induced to make a pattern with a size of 0.35 × 0.35 mm of plates for the capacitor. The patterned Cu plates were peeled off and covered both the top and bottom with PI film by a hot press under 400 K for 30 min. After the Cu plates were covered, PI was cut with a space of 0.1 mm. Then, PDMS was applied on the bottom Cu plate and aligned with the top plate. PDMS was cured under 60 °C until overnight. Capacitance versus pressure was measured with the force measurement instrument (Mark-10).

### Circuit Development

The stretchable interconnectors were modified from the previously developed structure. A 300 μm Cu foil was placed on the PDMS, and a femtosecond IR laser was induced to make a pattern with a width of 0.35 mm for the interconnector. The patterned Cu plates were peeled off and covered both top and bottom with PI film by the hot press under 400 K for 30 min. The PI film for the top cover had space for the connection with the sensors and fPCB. PI was cut with a space of 0.1 mm, and the excess film was removed. Prepared interconnects had different shapes to perfectly match with the glove. fPCB was designed by Altium. Components were placed on both the top and bottom to make a small size with a compact design. The fPCB was soldered in the laboratory with conductive solder paint and tacky flux under 180 °C.

### Glove System Integration

The pressure, temperature, and flexion strain sensors were first connected to the interconnectors and soldered to each connection point of the fPCB. The device was then connected to the rechargeable battery with a magnetic charge port. The battery was then fixed on the fPCB with PDMS and cured under 60 °C for 12 h, and the device, including interconnectors and sensors, was integrated with a glove with PDMS and cured; the wearable multimodal sensing glove was finally manufactured. The assembled wearable multimodal sensing glove was tested for the device’s performance. For the cyclability of the pressure sensing, the force measurement instrument was applied with repeating force. Each step was moved to a hammer with a distance between 0 and 2.5 mm to apply 100 N 100 times. The applied force from the instrument was compared with directly measured capacitance. A thermostat was used for temperature control to monitor the durability of the temperature. The temperature of the thermostat was raised up to 40 °C for 10 s and cooled down to 25 °C for 50 s. The temperature sensor of the glove was placed on the thermostat and repeated for 5 h. Finger flexion was directly measured with the glove in the same way as in the sensor test. The sensor was already validated, so only the motion angle of the index finger was captured from the video and compared with the strain sensor’s resistance change. All tests were measured from the device circuit of the glove and wirelessly transferred to the PC.

### Tactile Feedback System Integration

The band-shaped feedback system consisted of mechanical components, electronics, a microcontroller containing a control box, and different haptic feedback units integrated with a Velcro band. The control box was manufactured by assembly of 3D printed structures which are produced from a poly(lactic acid) printing filament, and its size was 8.45 (D) × 10.8 (W) × 21.6 (L) cm, which was designed to be small and lightweight for portability. The Arduino microcontroller, motors, syringes, 9 V battery slots, and connecting boards were integrated into the box. The motor was connected to a pinion and a rack that had a sawtooth to modulate the piston of the syringe. Those pinions and racks were fabricated from a 0.635 cm acrylic sheet using a CO_2_ laser instrument. And the motors were programmed to respond proportionately to the detected pressure from the glove. The Velcro band was connected to the control box via wires and tubes to operate feedback units. The band contained three proprioceptive feedback mechanisms: pressure, vibration, and displacement. Three Foley catheters were connected to 3 mL of syringes in the control box to give pressure feedback to control balloon expansion with inner tube pressure control. The vibrotactile temperature response was given by a commercially available vibrator based on PWM from 0 to 255. The vibration input intensity was modulated by the potential changes from 2 to 5 V, corresponding to the PWM. Two micro servomotors deliver the finger flexion response, and they are connected to a sliding band.

### Data Acquisition System

The integration of various sensors is key to the functionality of the wearable multimodal sensing glove. The glove’s analog-to-digital converters (ADC) accurately convert analog signals from various sensors into digital data that the NRF52832 microcontroller unit can process. The ADS1100 is a key component in the glove’s sensor array, functioning as a precision, low-power, channel converter. The FDC1004 is capable of converting capacitance measurements to digital values. Built-in active shield drivers effectively manage electromagnetic interference (EMI) for precise high-sampling readings. In addition, the glove also incorporates ADG728 and DAC5571, which measure parameters such as the temperature and strain. The integration of these sensors enables the glove to provide precise, real-time data acquisition, which is crucial for the effective rehabilitation of hand sensory impairments.

- MCU: NRF52832
- Sensors: TMP117, FDC1004, ADG728, DAC5571, ADS1100
- VCC voltage: 3.3 V
- Power consumption during sampling: 3.23 mW
- Expected battery life using a 70 mA h battery: 30.16 h

### Validation of the Tactile Feedback System

To confirm the tactile feedback system, the responses were tested with a wearable multimodal sensing glove. The glove was wirelessly connected to the Arduino system, and the feedback responses against the input signals were tested. For the pressure feedback, the changes in the pneumatic syringe for the mechanotactile response were compared with the detected sensory force from the glove. The force was given by manually pulling each finger, and it was measured using a force measurement instrument. An accelerometer measured the vibration acceleration of the vibrotactile response against a temperature change. The wearable multimodal sensing glove was placed on the 60 °C hot plate so the glove got heated, and the temperature sensor of the glove directly transferred the measured temperature to the feedback system to operate the vibrato. After that, the glove was placed on a 20 °C cooled glass plate. The vibrotactile response started decreasing vibration due to the decreased temperature of the glove. Finger flexion was confirmed with displacement changes by a hand gesture. The hand was manually changed, and then, the strain sensor of the glove gave feedback to move a band. Each strain sensor gave a 1/3 change of the displacement feedback system. The displacement was measured when only the thumb bent, the index and middle finger bent, and all fingers bent.

### Human Subject Study

To apply the sensing feedback glove in the actual life simulation protocol, an individual wore a wearable multimodal sensing glove on the left-hand and the Velcro band-shaped feedback system on the left arm. Then, the individual acts as an example by gripping a mug and pouring hot water, which is a reasonable situation in real life. To ensure that the feedback system changes, each step took enough time to give responses. First, the individual gripped a mug and kept it up for 20 s. Then, hot water was poured into the mug for 5 s, and the individual kept gripping it for about 35 s while increasing the temperature of the mug to vibrate the feedback system. After that, the individual puts down the mug to release all of the feedback systems. To take a supporting video for the operation of the feedback systems in the scenario, the feedback system was reversely worn on the left arm and repeated the same activity. All of the human pilot studies involved multiple healthy volunteers; the study followed the approved IRB protocol from the Georgia Institute of Technology (#H20457). All participants agreed to and signed the consent form to allow the experiment to proceed.

## Supporting Information Available

Wearable multimodal sensing feedback glove (PDF)
Operation of the pressure feedback (MP4)
Operation of the temperature feedback vibration response at the change of temperature (MP4)
Operation of the displacement response of the wearable multimodal sensing feedback glove with bending change of fingers (MP4)
Actual life scenario of the wearable multimodal sensing feedback glove (MP4)
Additional task scenarios of the wearable multimodal sensing feedback glove (MP4)

## Abbreviations

fPCB: flexible printed circuit board
LIG: laser-induced graphene
TSCI: traumatic spinal cord injury
SEM: scanning electron microscopy
VR: virtual reality
PWM: pulse-width modulation
