# Supplementary material for: Soft Nanomembrane Sensor-Enabled Wearable Multimodal Sensing and Feedback System for Upper-Limb Sensory Impairment Assistance
Source: ACS Nano. 2025 Jan 31;19(5):5613–28. doi: 10.1021/acsnano.4c15530 (PMC11823636; doi:10.1021/acsnano.4c15530)
Supplement: Supplementary file 1 — nn4c15530_si_001.pdf [file nn4c15530_si_001.pdf]

Supporting Information

Soft Nanomembrane Sensor-Enabled Wearable Multimodal Sensing and Feedback System for Upper-Limb Sensory Impairment Assistance

Tae Woog Kang,[a,b,†] Yoon Jae Lee,[a,c,†] Bruno Rigo,[a,c] Ira Soltis,[a,b] Jimin Lee,[a,b] Hodam Kim,[a,b] Gaorong Wang, [a,c] Nathan Zavanelli, [a,c] Eyas Ayesh,[d] Wali Sohail,[d] Houriyeh Majditehran,[d] Scott H. Kozin,[e] Frank L. Hammond III,[b,d,f,*] and Woon-Hong Yeo [a,b,f,g,*]

[a] Wearable Intelligent Systems and Healthcare Center (WISH Center), Institute for Matter and Systems, Georgia Institute of Technology, Atlanta, GA 30332, USA
[b] George W. Woodruff School of Mechanical Engineering, Georgia Institute of Technology, Atlanta, GA 30332, USA
[c] School of Electrical and Computer Engineering, Georgia Institute of Technology, Atlanta, GA 30332, USA
[d] Adaptive Robotic Manipulation Laboratory, George W. Woodruff School of Mechanical Engineering, Georgia Institute of Technology, Atlanta, GA 30332, USA
[e] Shriners Hospital for Children, Philadelphia, PA 19140, USA
[f] Wallace H. Coulter Department of Biomedical Engineering, Georgia Institute of Technology and Emory University School of Medicine, Atlanta, GA 30332, USA
[g] Parker H. Petit Institute for Bioengineering and Biosciences, Institute for Robotics and Intelligent Machines, Georgia Institute of Technology, Atlanta, GA 30332, USA
[†]These authors contributed equally to this work
* To whom correspondence should be addressed: Prof. Woon-Hong Yeo: whyeo@gatech.edu or Prof. Frank Hammond: frank.hammond@me.gatech.edu

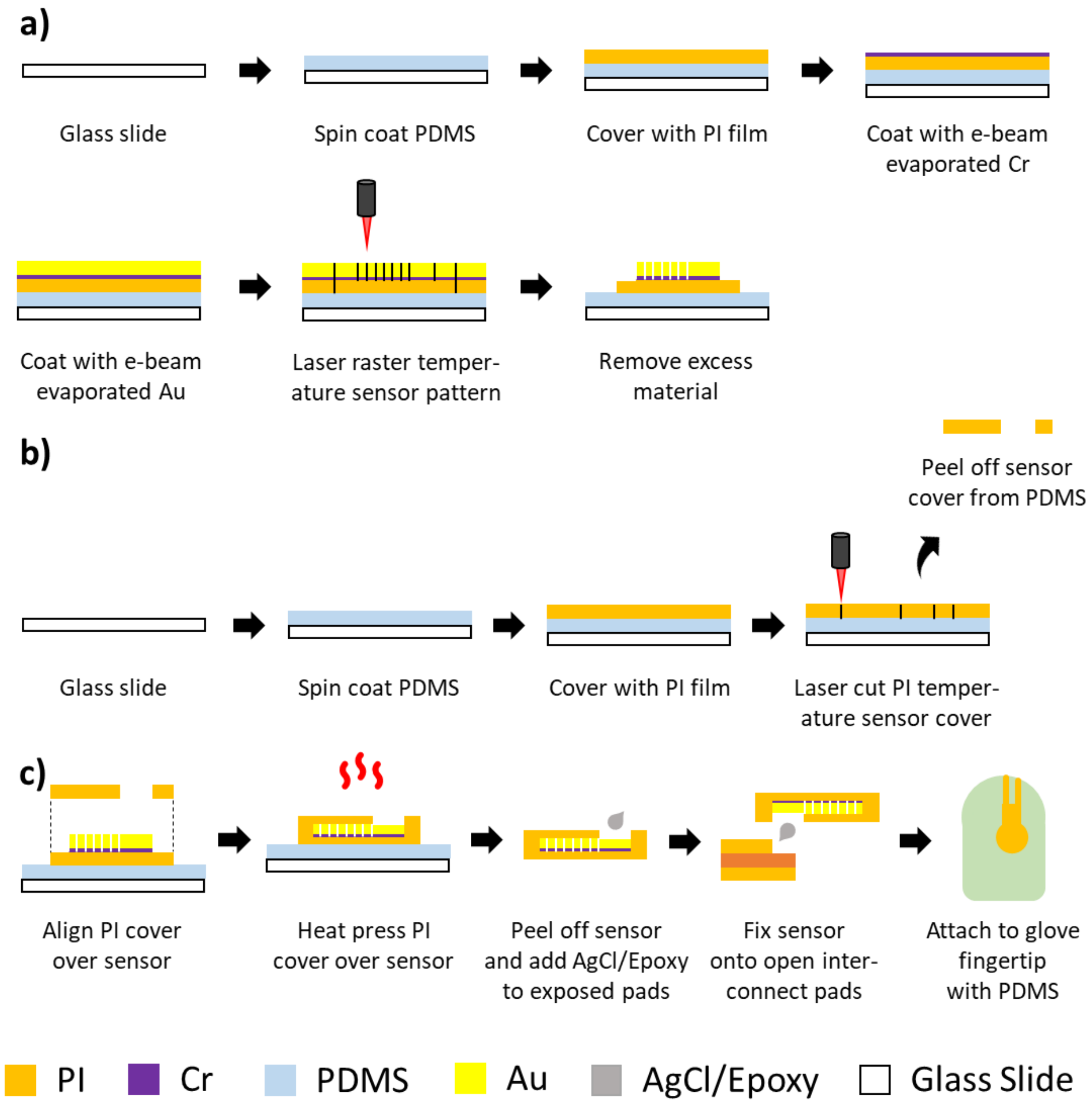

Figure S1. (a) Schematic illustration of the fabrication procedure for the hypnotic spiral Au structure, **(b)** PI sensor cover on the gold, and **c)** assembly step for the temperature sensor.

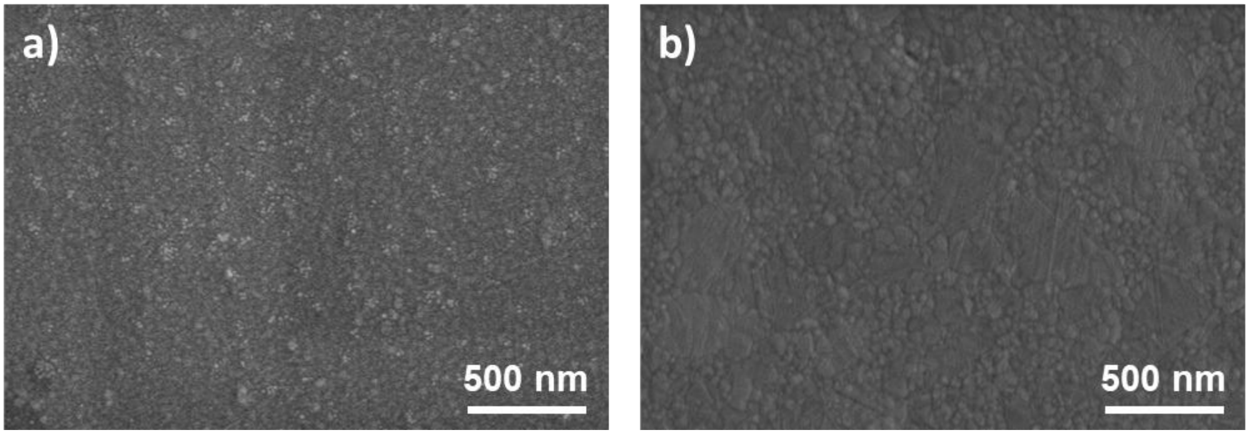

Figure S2. (a) SEM top-view image of Au plate with different 50 nm and (b) 100 nm thicknesses.

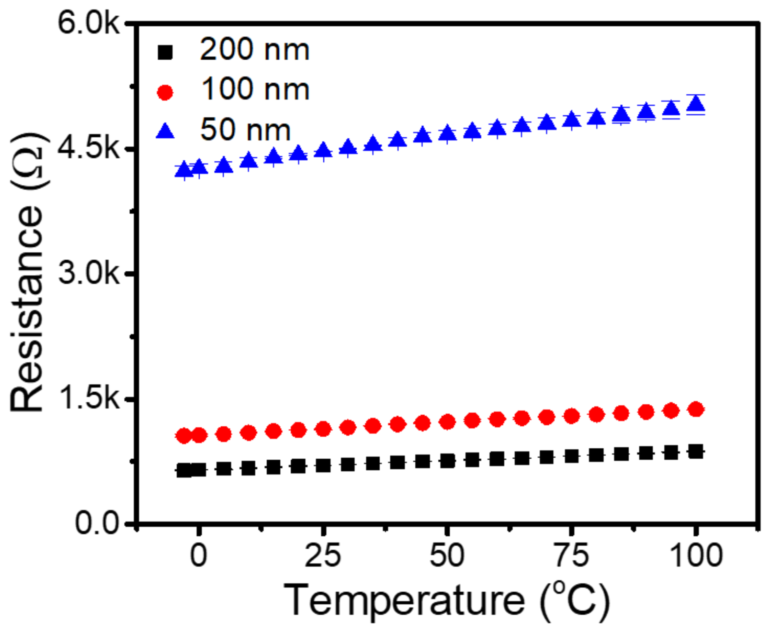

Figure S3. Resistance of the temperature sensor according to temperature changes with different gold thicknesses.

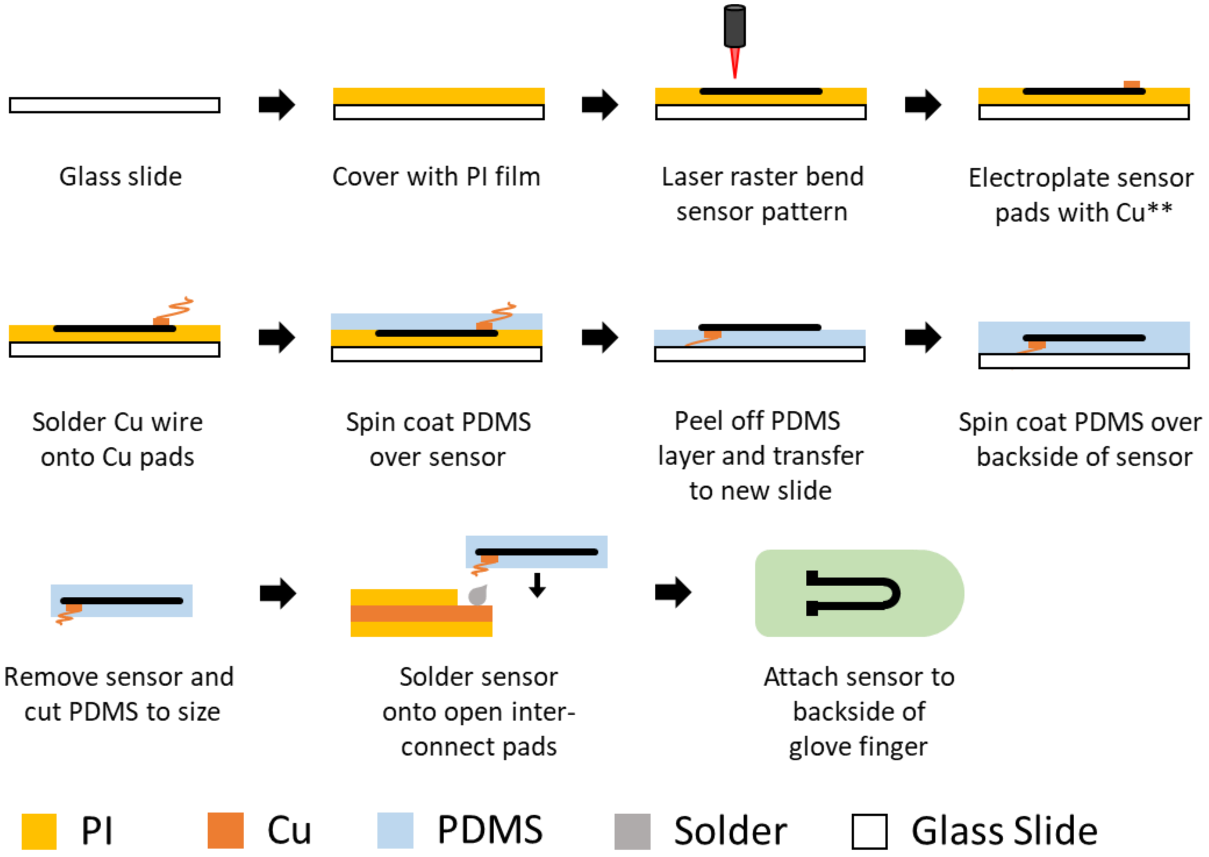

Figure S4. Schematic illustration of the fabrication procedure for the laser-induced graphene-based strain sensor.

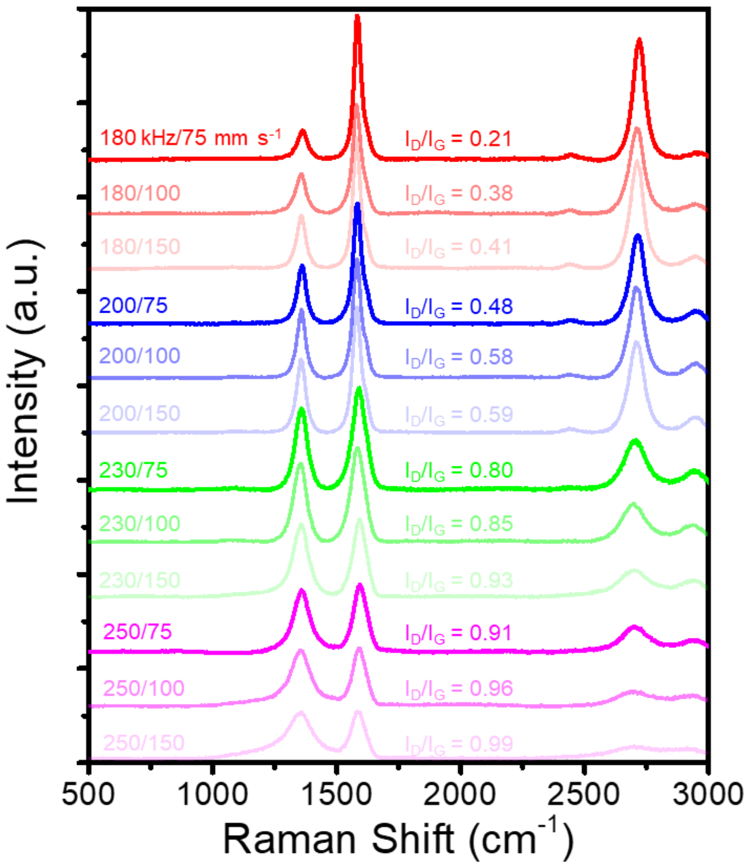

Figure S5. Raman spectra and graphene D/G peak ratio of the laser-induced graphene with different laser frequencies and scan rates.

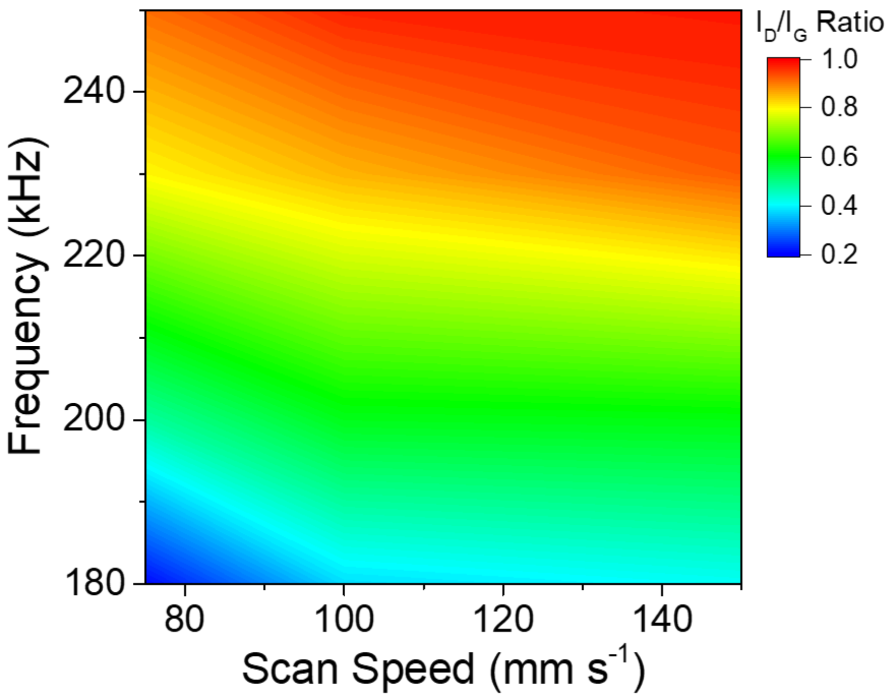

Figure S6. 2D contour mapping of I_D/I_G peak ratio of laser-induced graphene with different scan speeds and frequency conditions.

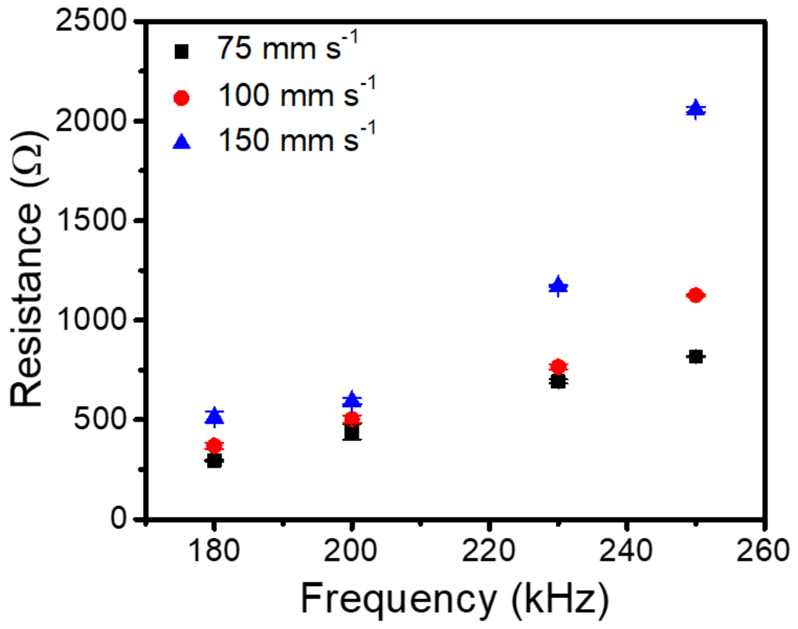

Figure S7. Resistance of pristine laser-induced graphene on PI film with different scan speeds and frequency conditions.

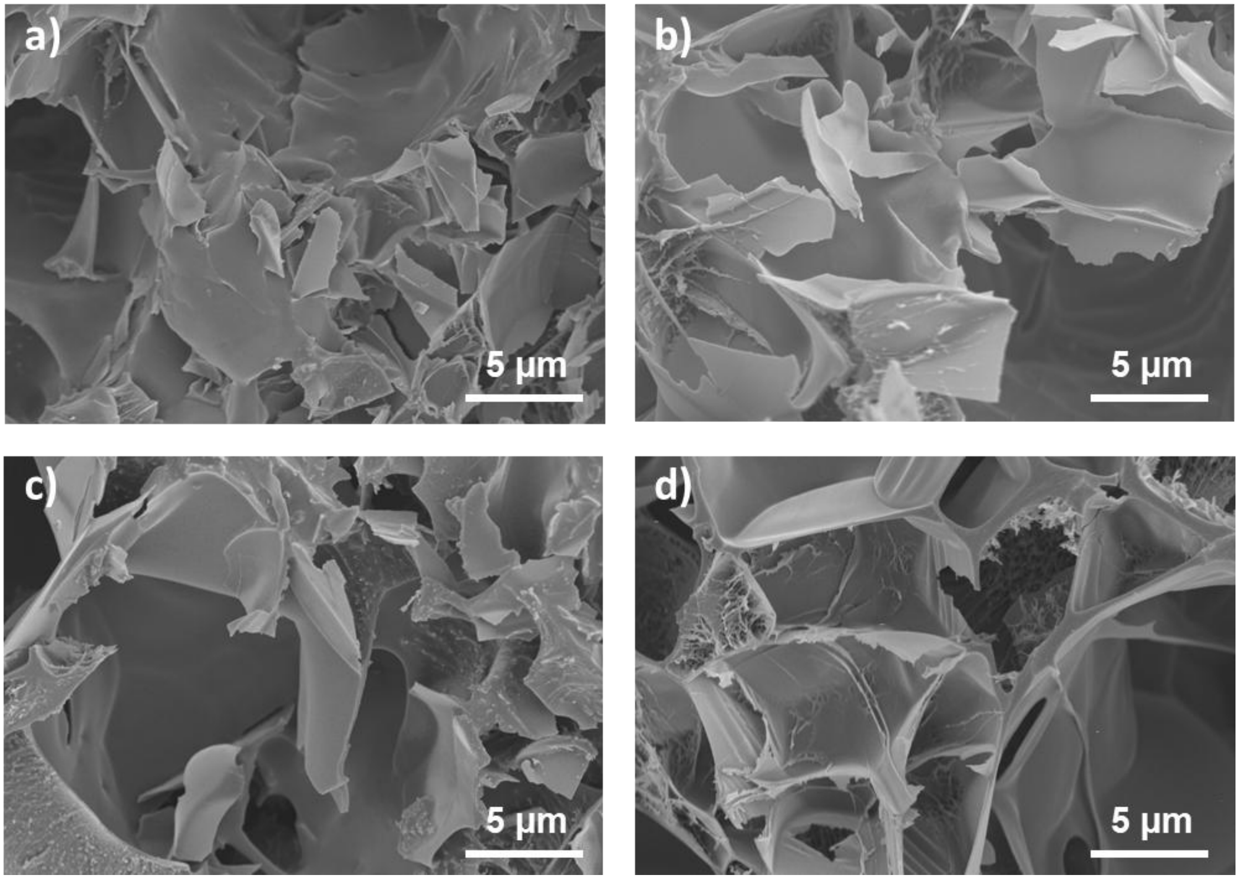

Figure S8. Top-view SEM image of the laser-induced graphene with different laser scribing conditions. **(a)** Laser-induced graphene with 180 kHz laser frequency and 150 mm s^{-1} scan speed, **(b)** 200 kHz and 75 mm s^{-1}, **(c)** 230 kHz and 75 mm s^{-1}, and **(d)** 250 kHz and 75 mm s^{-1}.

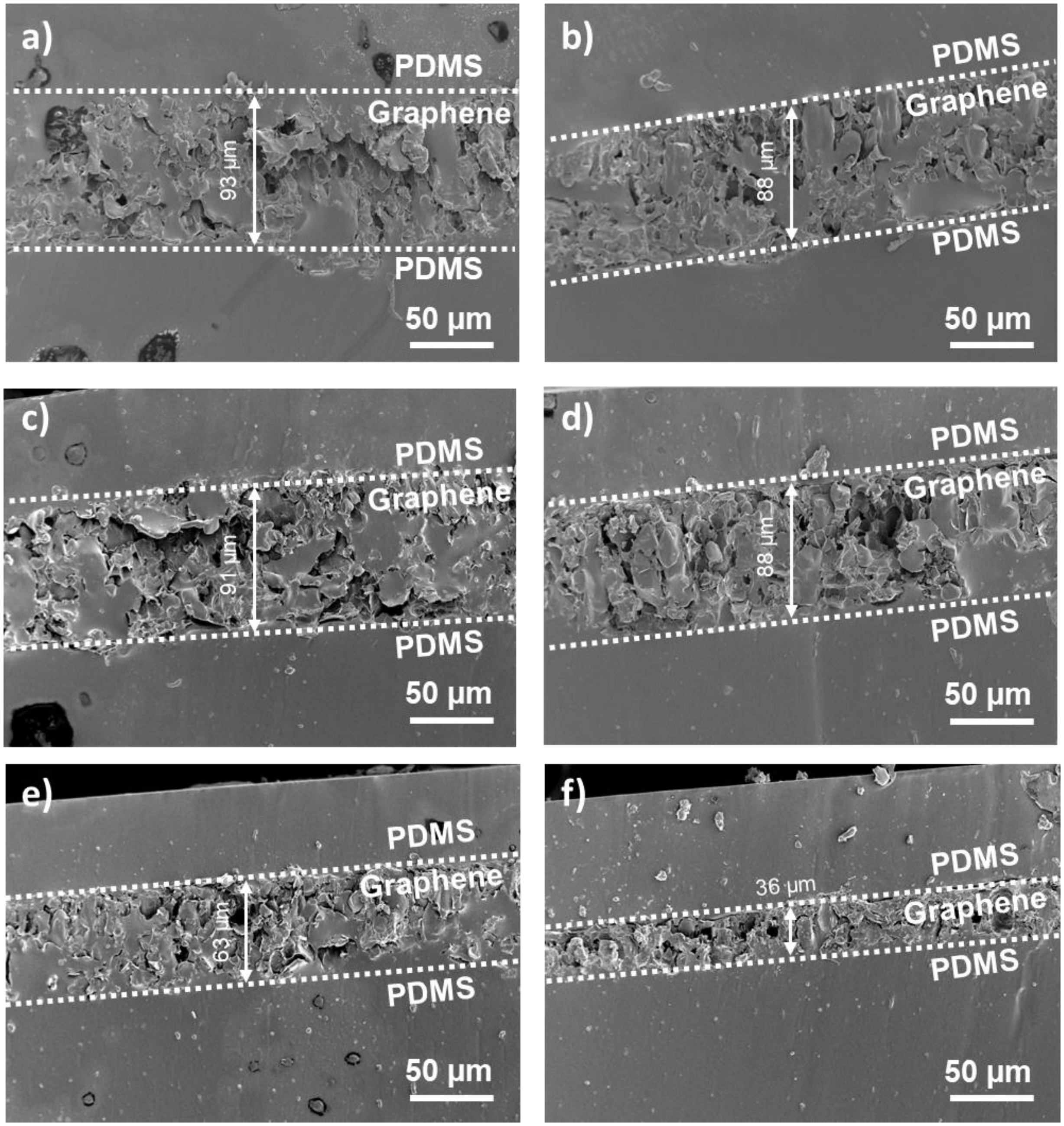

Figure S9. Vertical section SEM image of the laser-induced graphene with different laser scribing conditions. **(a)** Laser-induced graphene with 180 kHz laser frequency and 100 mm s^{-1} scan speed, **(b)** 180 kHz/150 mm s^{-1}, **(c)** 200 kHz/75 mm s^{-1}, **(d)** 230 kHz/75 mm s^{-1}, **(e)** 250 kHz/75 mm s^{-1}, and **(f)** 250 kHz/150 mm s^{-1}.

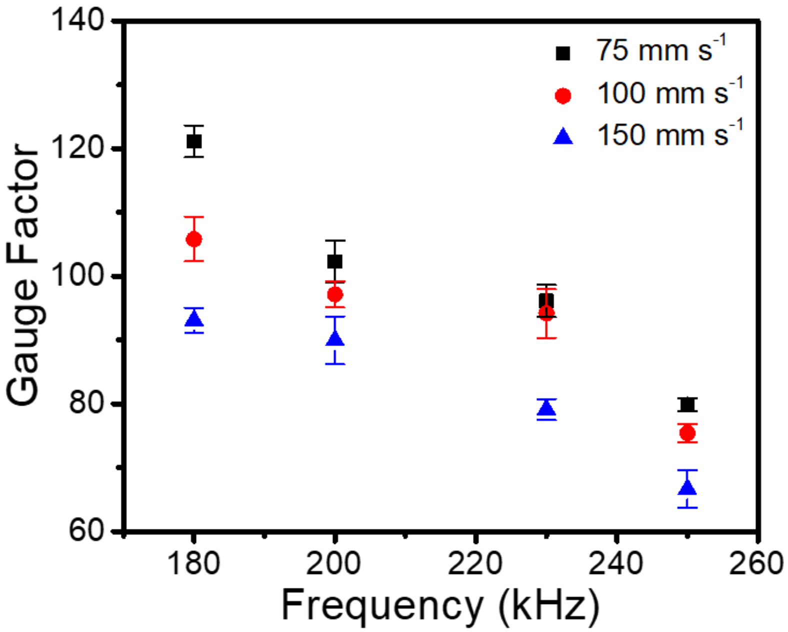

Figure S10. Gauge factor of laser-induced graphene with different scan speeds and frequency conditions.

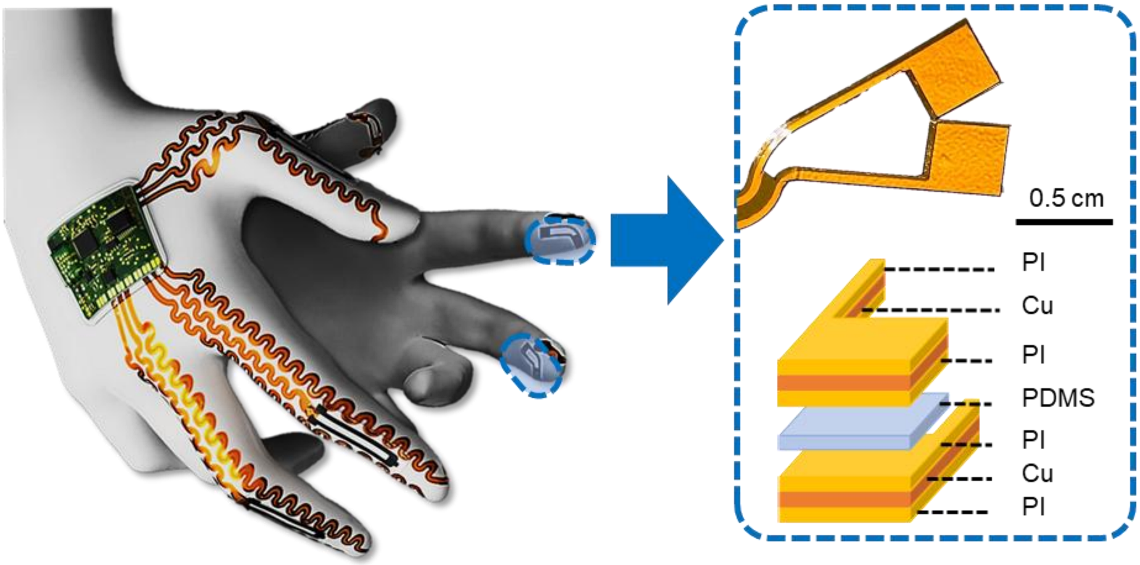

Figure S11. Illustration of a wearable multimodal sensing glove and the detailed structure of a capacitance pressure sensor.

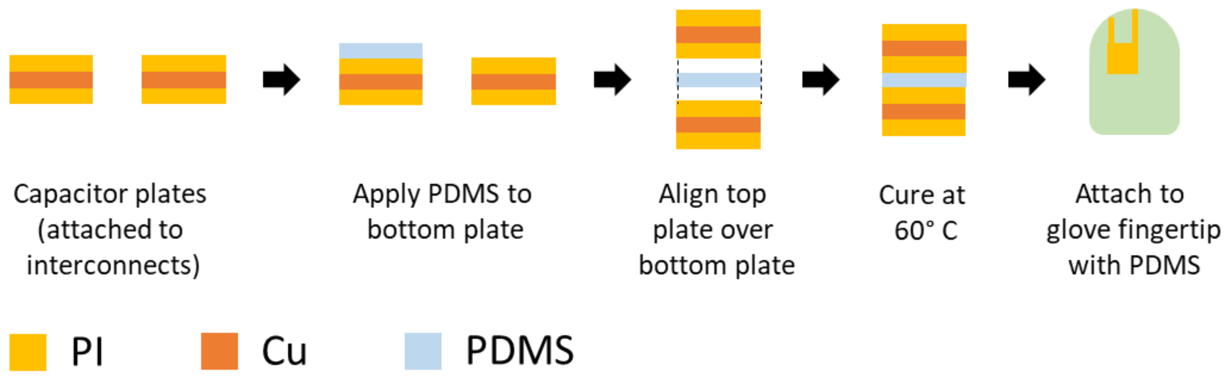

Figure S12. Schematic illustration of the fabrication procedure for the capacitance pressure sensor.

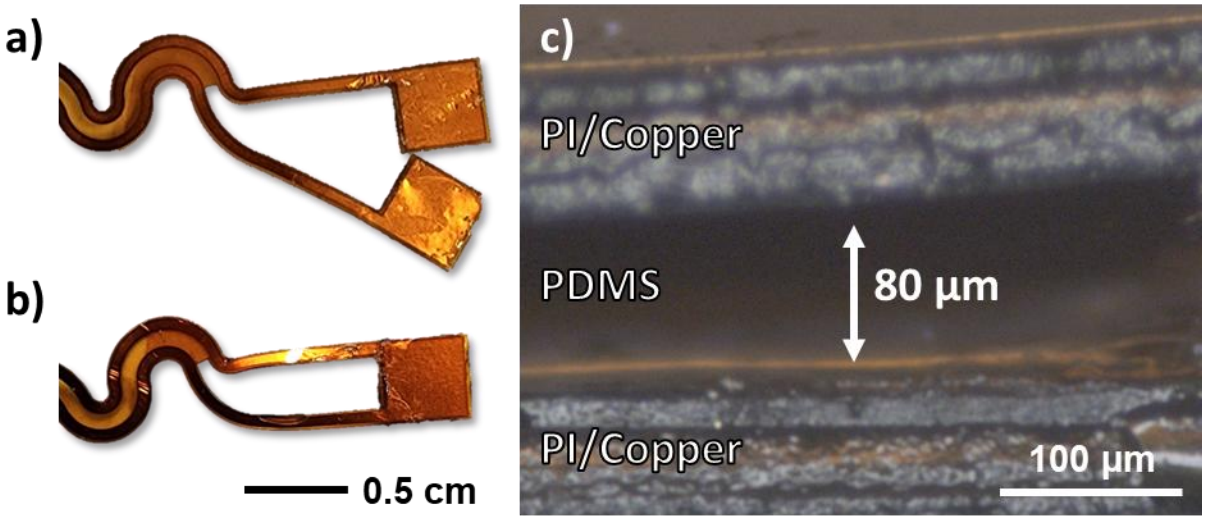

Figure S13. (a) Optical photograph of the pressure sensor before and **(b)** after assembly with PDMS dielectric layer. Schematic illustration of the fabrication procedure for the capacitance pressure sensor. **(c)** Vertical section image after assembly of the pressure sensor.

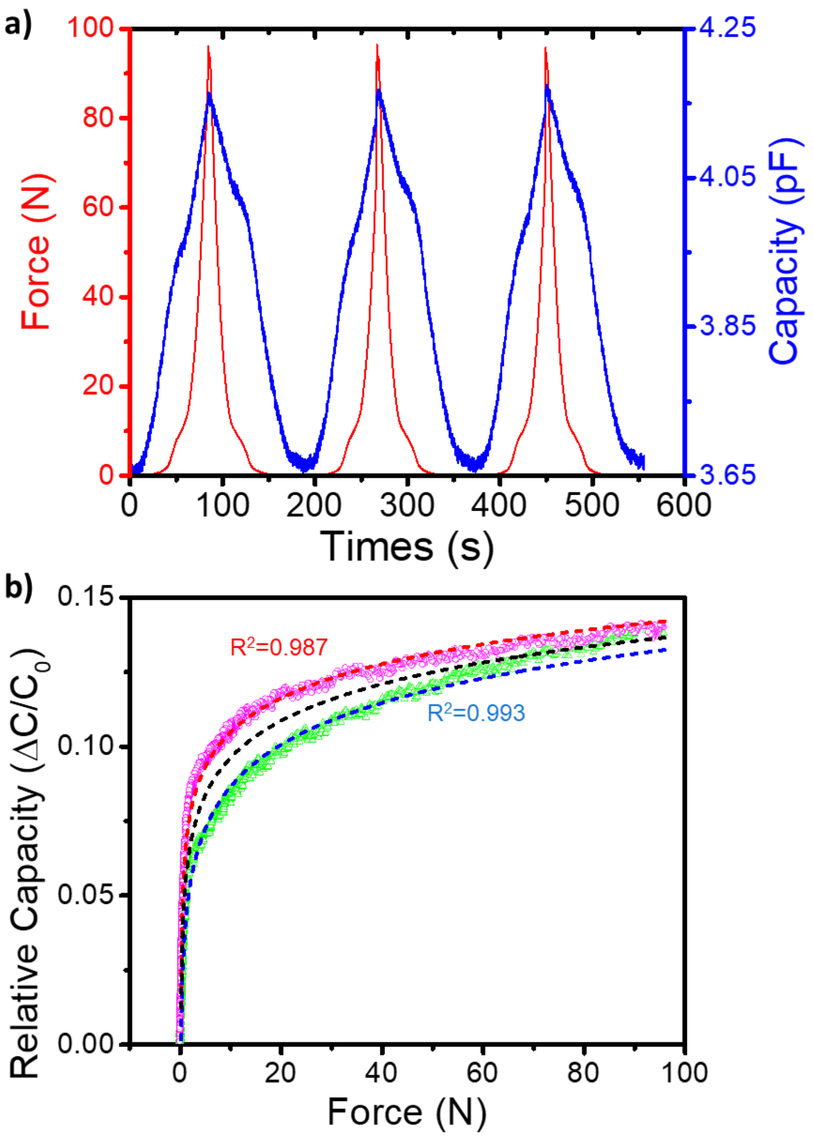

Figure S14. (a) Capacitance pressure sensor responses upon loading and unloading force and **(b)** hysteresis graph.

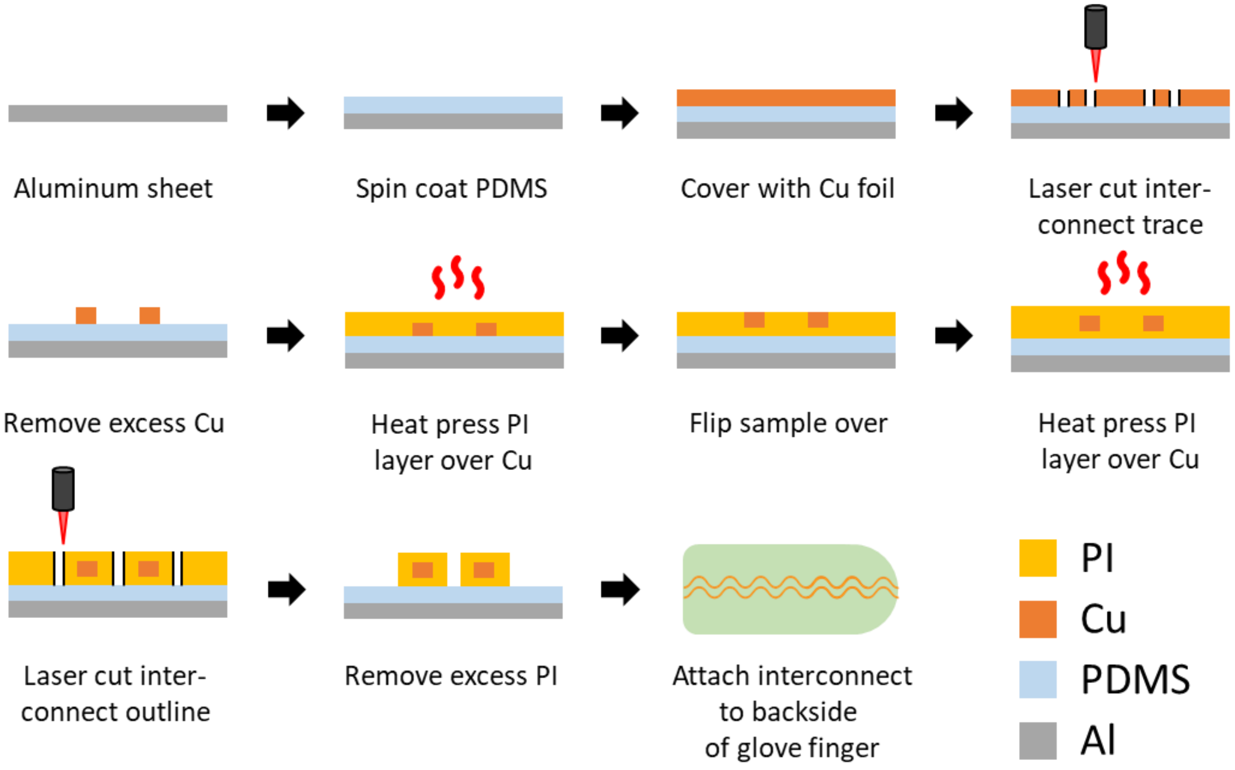

Figure S15. Schematic illustration of the fabrication procedure for the interconnectors

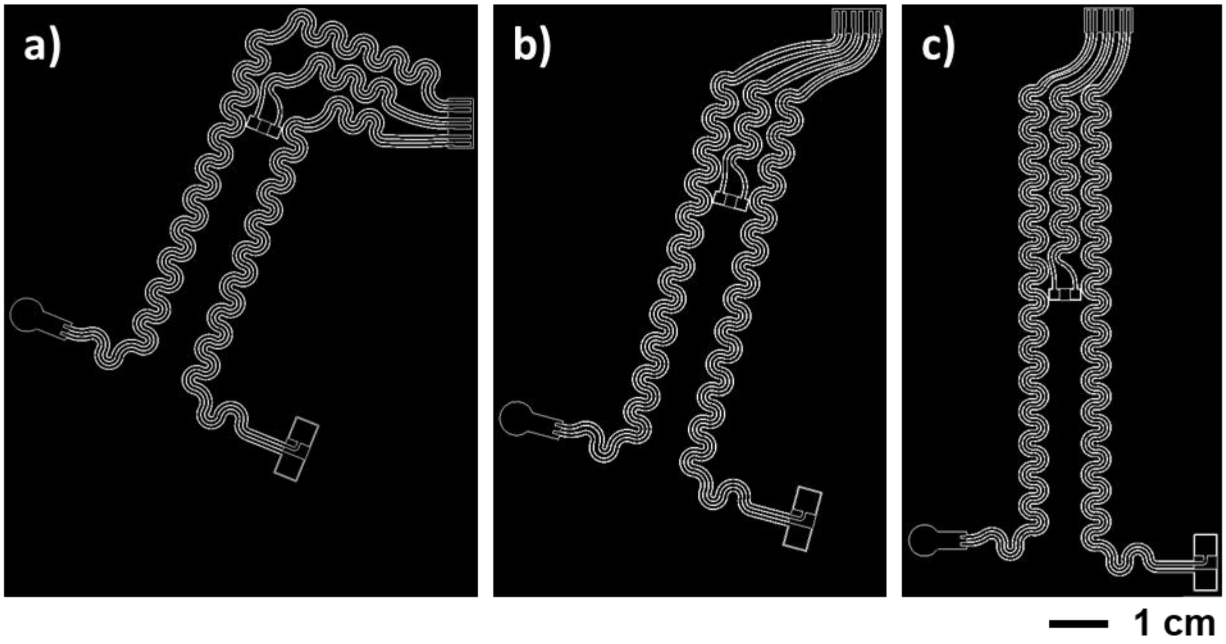

Figure S16. Schematic CAD design of the interconnectors for the **a)** thumb, **b)** index finger, and **c)** middle finger.

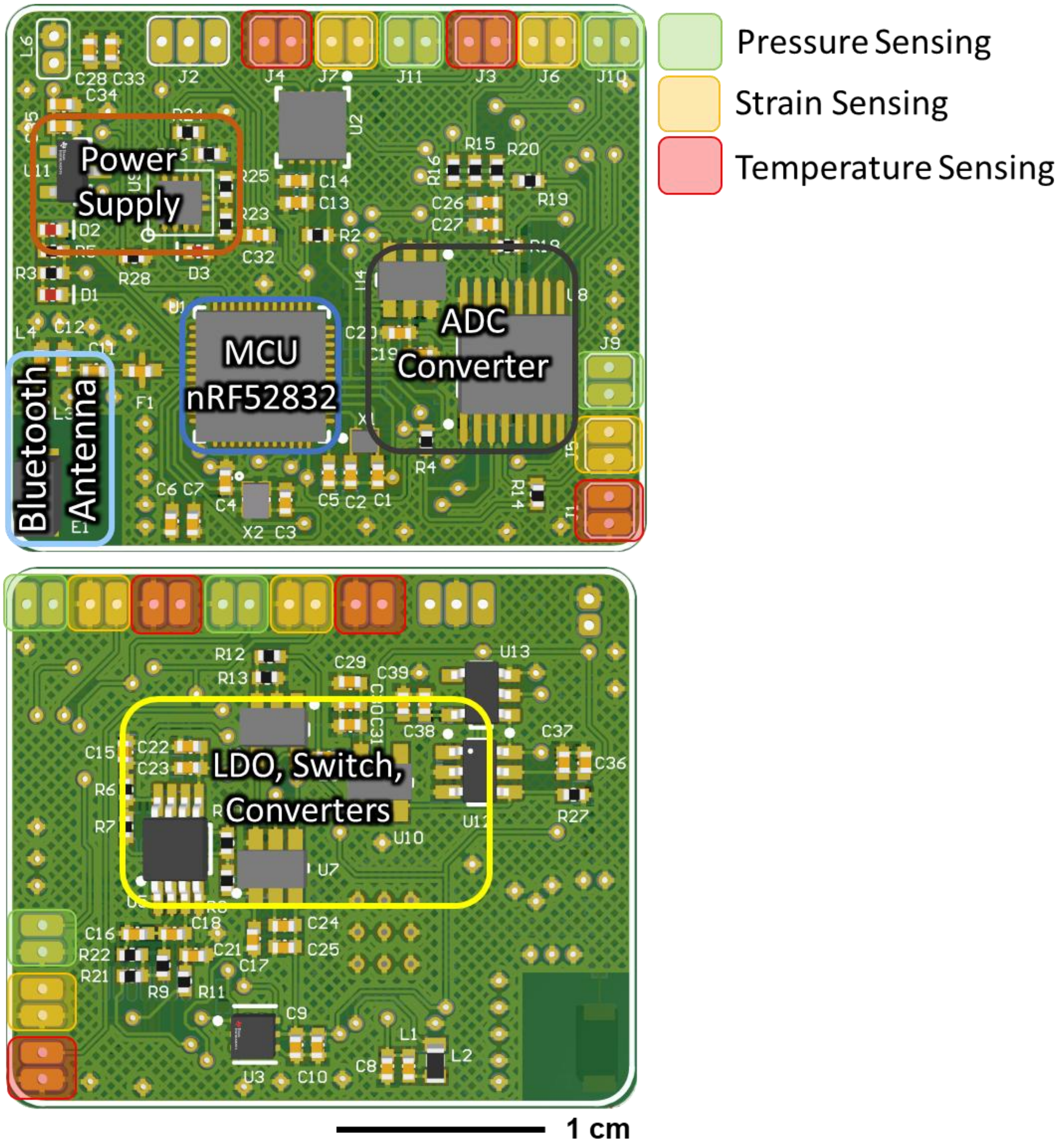

Figure S17. Flexible circuit design of wearable sensing grove that measures ambient and contact temperature, pressure, and strain.

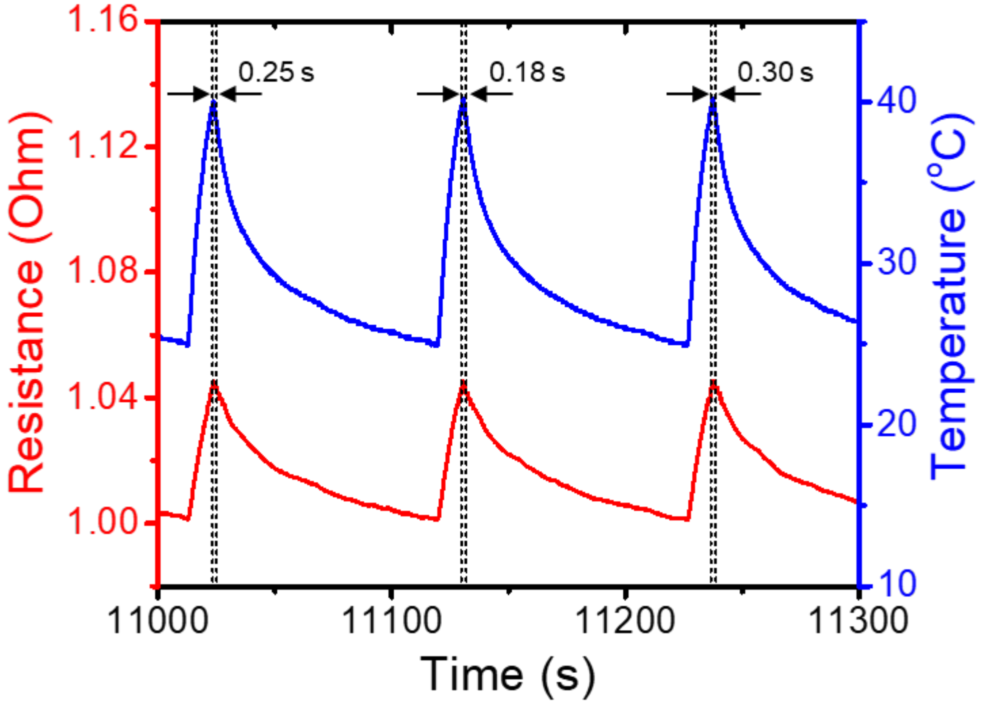

Figure S18. The response delay of the temperature sensor under temperature changes from 40 ^{o}C to 25 ^{o}C.

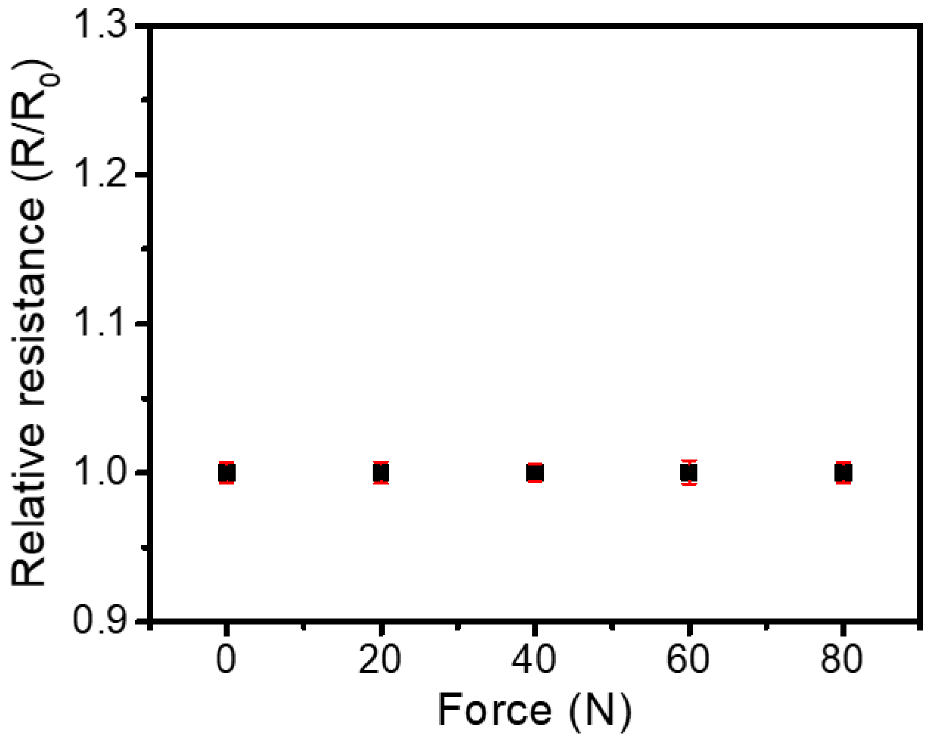

Figure S19. Responses of the temperature sensor regarding loading different pressures.

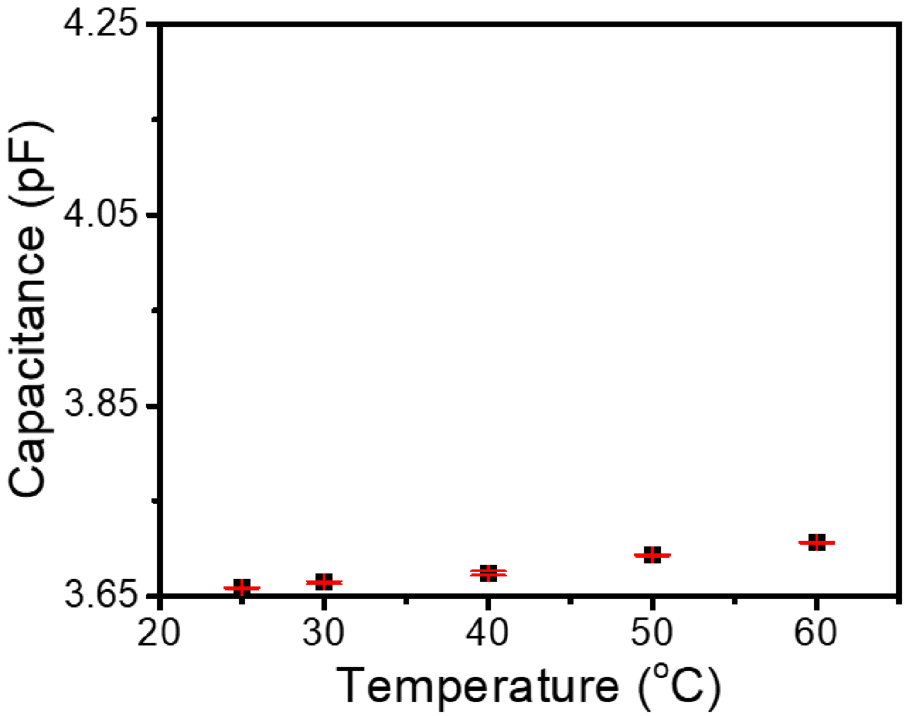

Figure S20. Capacitance responses of the pressure sensor under different temperatures.

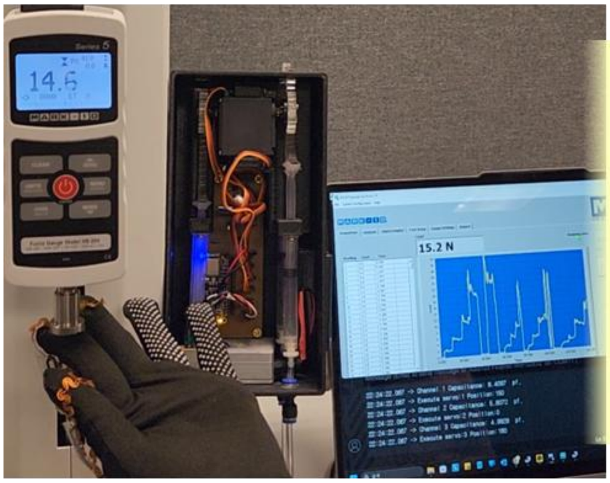

Stage	Time
Sensor Sensing	< 250 ms
Signal Processing	< 20 ms
Transmission	Negligible to 10 ms for wireless
Arduino Processing	20 - 40 ms
Triggering Actuation	10 - 20 ms
Feedback Loop	< Every 333 ms

Figure S21. Elapsed time from sensing to feedback execution

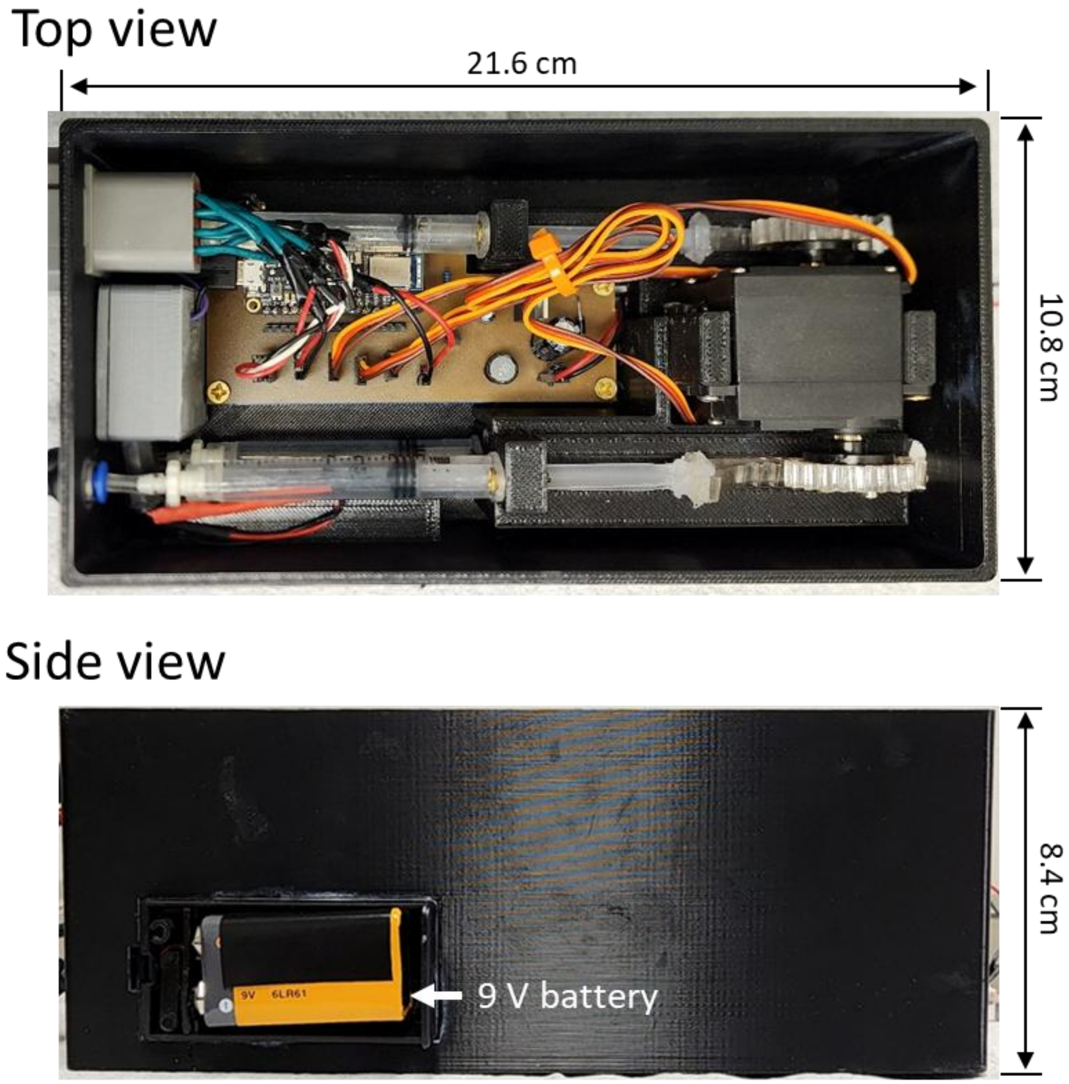

Figure S22. Top and side view of the assembled feedback control box.

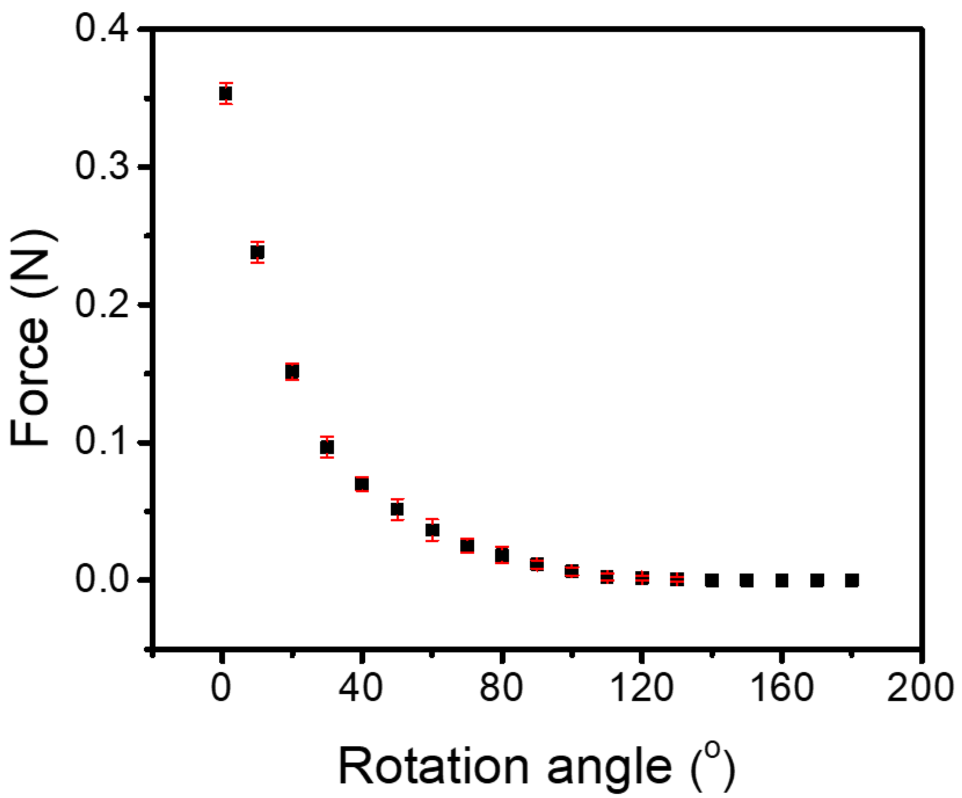

Figure S23. The response force of the mechanotactile pressure feedback with a manually rotated motor angle.

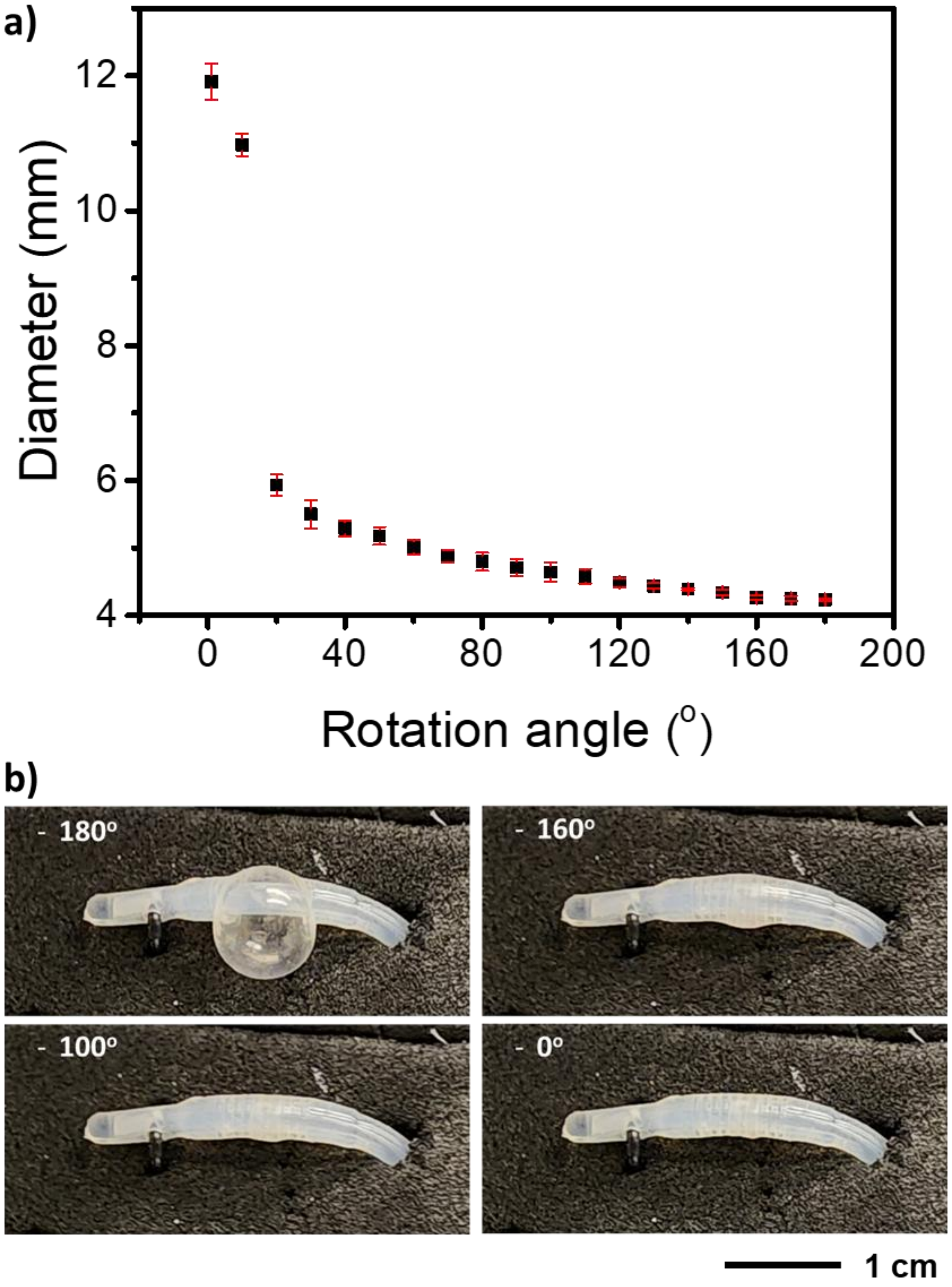

Figure S24. (a) Diameter change of the balloon of mechanotactile pressure feedback with manually rotated motor angle and **(b)** photographic image of the balloon with different input angle.

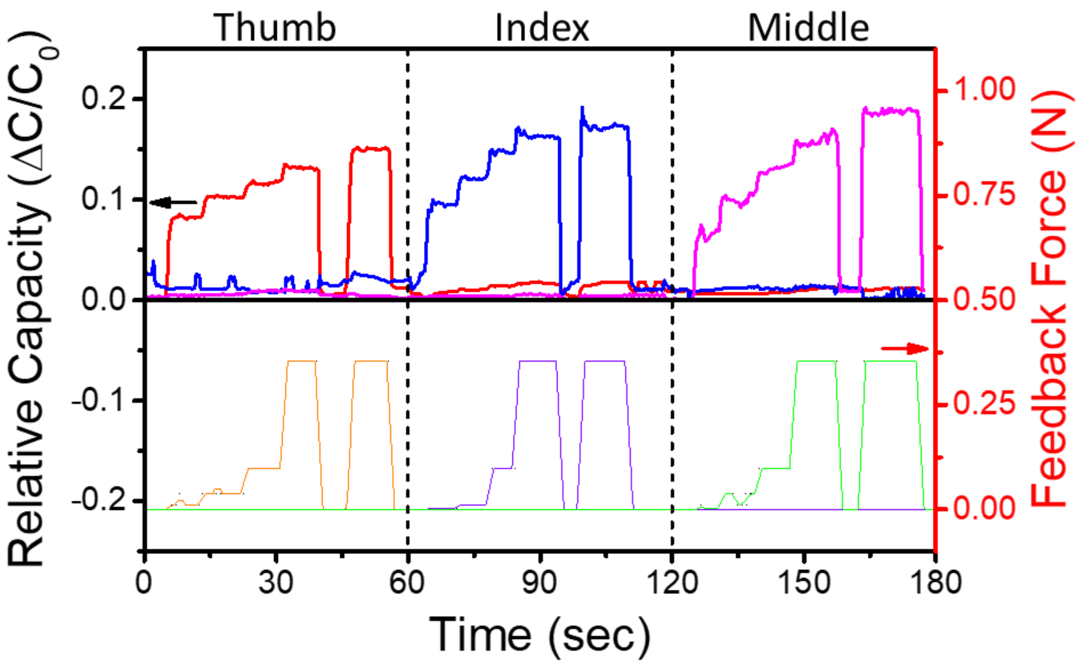

Figure S25. Relative capacity change of the pressure sensor and responded feedback force during the pressure feedback response test.

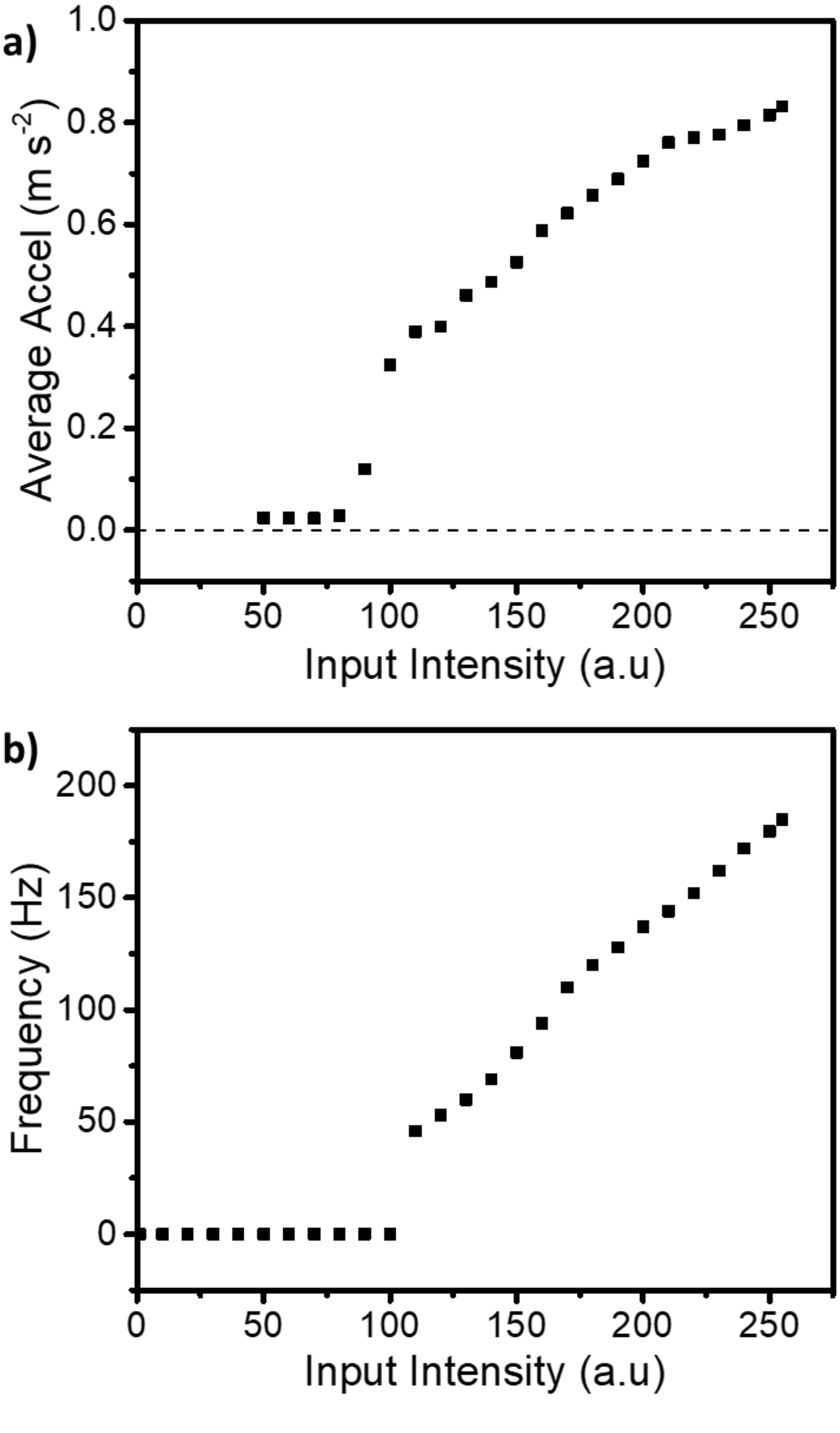

Figure S26. (a) Vibrate acceleration changes of the vibrotactile temperature feedback with manual input intensity and **(b)** its frequency change of the vibration sound.

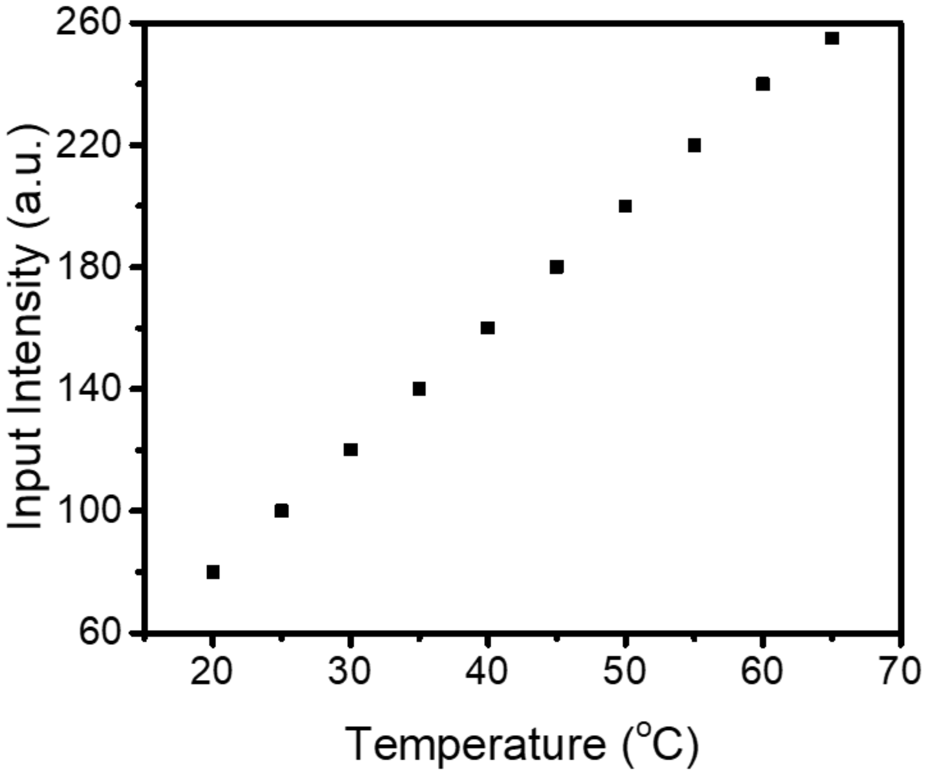

Figure S27. Manual input intensities of the vibrotactile feedback under varying temperatures from 20 °C to 65 °C.

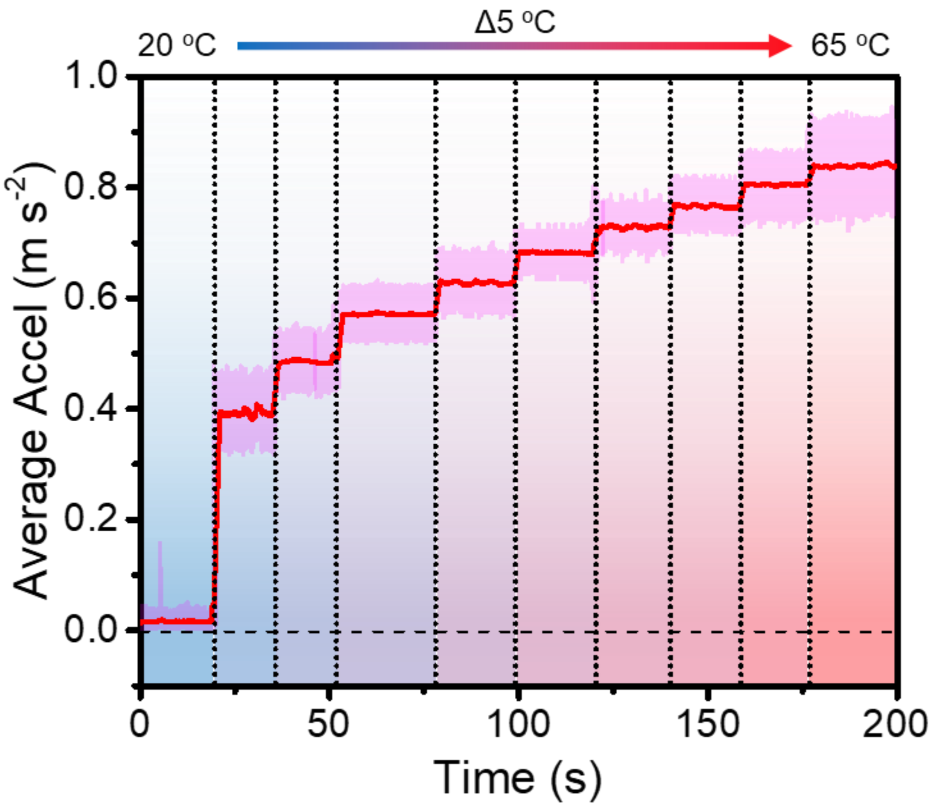

Figure S28. Vibrate acceleration response of the vibrotactile feedback with temperature changes from 20 ºC to 65 ºC with 5 ºC intervals.

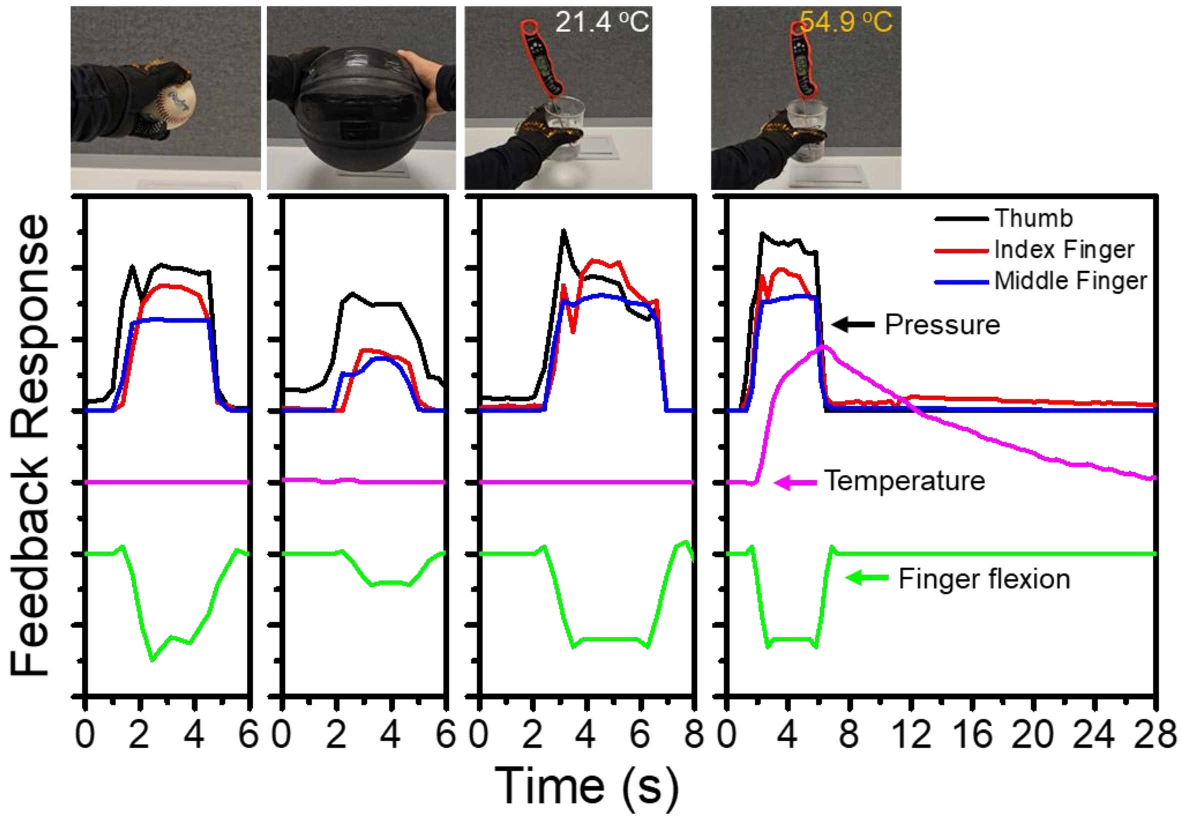

Figure S29. Demonstration of the wearable multimodal sensing and feedback glove with different task scenarios: gripping a baseball, a basketball, a cold beaker, and a hot beaker.

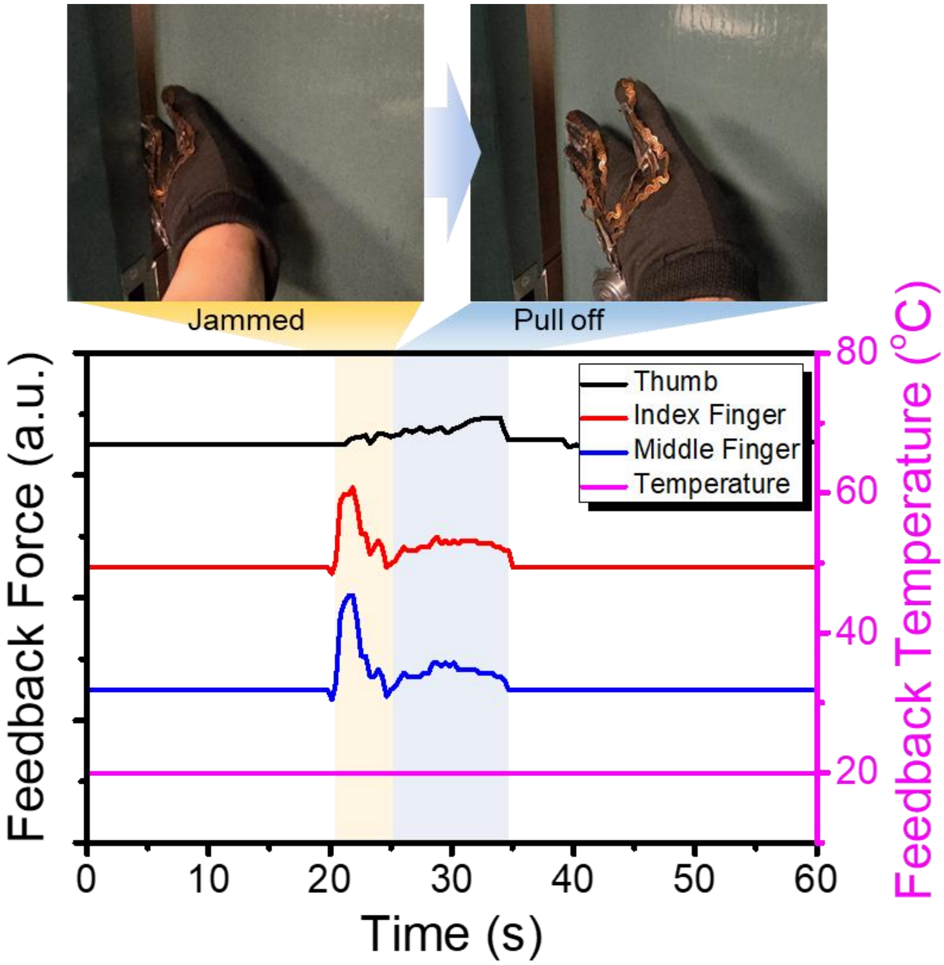

Figure S30. Demonstration/Application of the wearable multimodal sensing feedback glove responding to pressure hazards caused by a door jamming.

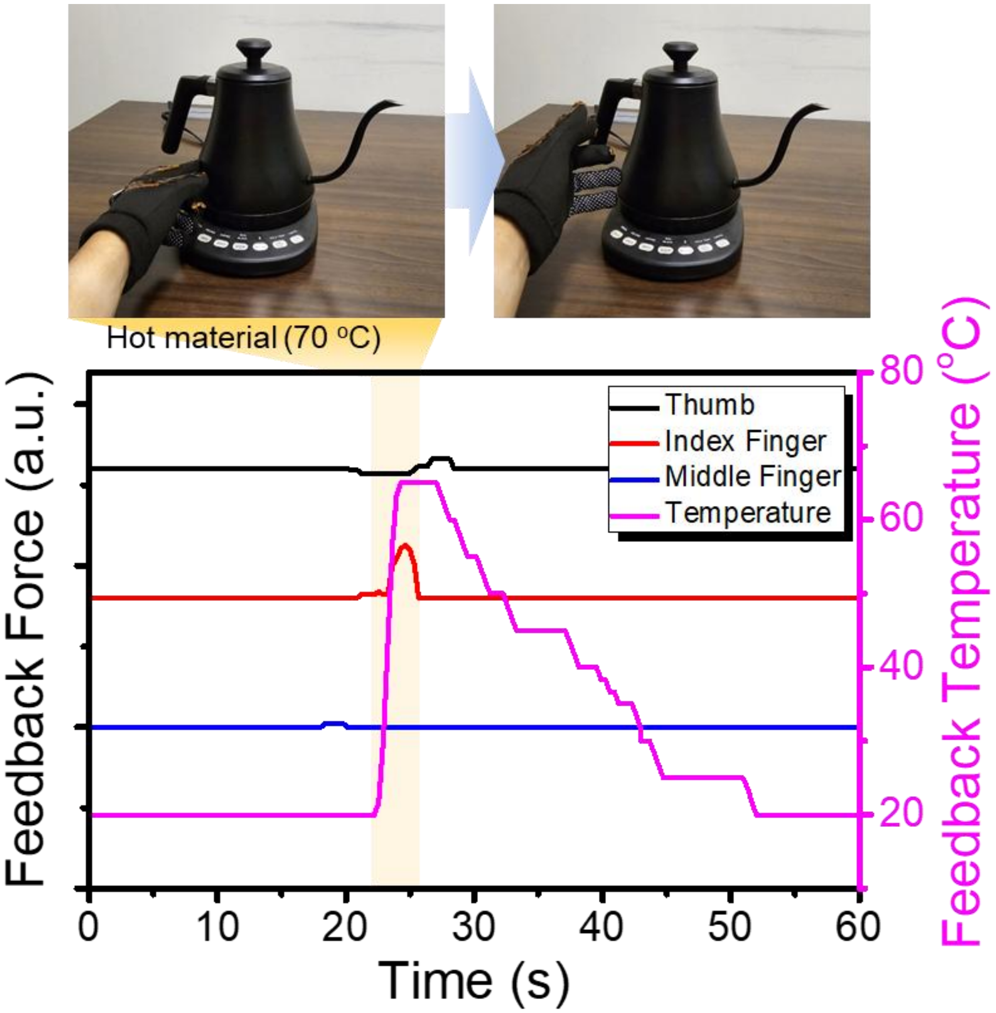

Figure S31. Demonstration/Application of the wearable multimodal sensing feedback glove responding to thermal hazards when touching a hot kettle (70 °C).

Table S1. Comparison of the wearable multimodal sensing feedback glove with previously reported upper-limb sensory impaired patient assistance systems.

Reference	Sensing Device	Sensory Part	Sensor Property			Feedback System		
			Pressure	Temperature	Flexion	Pressure	Temperature	Flexion
This paper	Glove	Pressure, Strain, Temperature	0.006 (pF/N)	2.10 (Ohm/ºC)	37 (Ohm/deg)	Mechanotactile pressure	Vibrotactile	Mechanotactile shearing
de Paz *et al.* (2023)[1]	Glove	Pressure	-	-	-	Vibrotactile, Acoustic	-	-
Abdelrahman *et al.* (2021)[2]	Glove	Pressure	-	-	-	Acoustic	-	-
Clemente *et al.* (2016)[3]	Thimble	Pressure	0.2 (V/N)	-	-	Vibrotactile	-	-
Semere *et al.* (2015)[4]	Glove	Pressure	-	-	-	Vibrotactile	-	-
Hafidh *et al.* (2013)[5]	Glove	Pressure	8 (mS/N)	-	-	Acoustic	-	-
Mendes *et al.* (2011)[6]	Glove	Pressure	-	-	-	Acoustic	-	-

Table S2. Comparison of micro hypnotic spiral-patterned gold disc with previously reported wearable temperature sensors.

Material	Measurement Range	TCR	R-squared	Reference
**Au/Cr/PI**	**-3 ~ 100 ^{o}C**	**0.0032 ($^{o}C^{-1}$)**	**0.999**	**This paper**
rGO fiber	30 ~ 80 ^{o}C	0.0019 (K^{-1})	-	7
Graphene/PI	20 ~ 50 ^{o}C	0.0085 ($^{o}C^{-1}$)	0.98	8
PEDOT:PSS-PDMS	25~ 40 ^{o}C	0.0031 ($^{o}C^{-1}$)	0.991	9
CNT/PEDOT:PSS	25 ~ 50 ^{o}C	- 0.0031 ($^{o}C^{-1}$)	0.97	10
PEDOT-TPU fiber	5 ~ 40 ^{o}C	0.0093 ($^{o}C^{-1}$)	0.998	11
MWCNT-PET	30 ~ 60 ^{o}C	- 0.000685 ($^{o}C^{-1}$)	0.99	12
Ag-MWCNT/PEDOT:PSS	25 ~ 80 ^{o}C	- 0.0045 ($^{o}C^{-1}$)	0.998	13

Table S3. Comparison of laser-induced graphene with previously reported wearable strain sensors with low applied Strain.

Material	Sensor Type	Applied Strain	Gauge Factor	Reference
**LIG/PDMS**	**Resistive**	**0 ~ 20 %**	**121**	**This paper**
Me-DLC	Resistive	0 ~ 60 %	2 ~ 12	14
GF/PDMS	Resistive	0 ~ 70 %	29	15
Graphite/Silk	Resistive	0 ~ 15 %	14.5	16
CNTs-PA6/PVA	Resistive	0 ~ 200 %	11.7	17
CB/PDMS	Resistive	0 ~ 10 %	15.75	18
CB/AgNPs	Resistive	0 ~ 100 %	21.12	19
PDA/CNT/EB	Resistive	0 ~ 250 %	5.06	20
PDMS/Graphite	Resistive	0 ~ 70 %	37	21
Spring carbon yarn	Resistive	3 ~ 25 %	121	22
Ag/PDMS	Resistive	0 ~ 32 %	9	23
ENIG/NBR	Resistive	0 ~ 40 %	62	24
TPUEM/CNTs/AgNPs	Resistive	0 ~ 250 %	9.6	25
Conductive yarn	Resistive	0 ~ 40 %	1.19	26
TPU/PDA/MXene	Resistive	0 ~ 75 %	21.73	27
CNT/PDMS	Capacitive	0 ~ 200 %	1	28
VACNT/PDMS	Capacitive	0 ~ 1 %	0.637	29

Table S4. Comparison of the wearable multimodal sensing glove with previously reported sensing gloves.

	**Sensor Target**	**Detection Range**	**Weight**	**Flexibility***	**Cost****
**This paper**	**Pressure**<br>**Temperature**<br>**Finger flexion**	**95 N**<br>**20~65 ºC**<br>**GF 121**	**18 g**	**Good**	**$ 13.4**
Duan *et al.* (2023)[30]	Finger flexion	GF 29	26 g	Good	$ 13.5
Au *et al.* (2023)[31]	Finger flexion	-	45 g	Poor	$ 52
Duan *et al.* (2022)[32]	Finger flexion	GF 10	50 g	Good	$ 34
Deng *et al.* (2022)[33]	Pressure<br>Finger flexion	15 N<br>-	161 g	Poor	$ 52
Oh *et al.* (2021)[34]	Temperature	35-55 ºC	-	Poor	$ 52
Chen *et al.* (2021)[35]	Pressure<br>Finger flexion	15 N<br>-	100 g	Bad	$ 100
Hosseini *et al.* (2018)[36]	Force	80 N	360 g	Bad	$ 100
Choi *et al.* (2018)[37]	Inertial	-	70 g	Bad	$ 40
Ryu *et al.* (2008)[38]	Pressure	12 N	1020 g	Bad	$ 100

* Good – All components (sensors, interconnectors, and circuit board except for a battery) are flexible; Poor – Some components (Two components of them) are flexible; Bad – One of them or none of them are flexible.

** For the unknown price of the wearable sensing glove in the reference, the cost was estimated based on the price of gloves, materials, and circuit board.

References

(1) de Paz, C.; Travieso, D. A direct comparison of sound and vibration as sources of stimulation for a sensory substitution glove. *Cogn Res Princ Implic* **2023**, *8* (1), 41. DOI: 10.1186/s41235-023-00495-w

(2) Abdelrahman, Y.; Bennington, M.; Huberts, J.; Sebt, S.; Talwar, N.; Cauwenberghs, G. Sensory Substitution for Tactile Feedback in Upper Limb Prostheses. *Annu Int Conf IEEE Eng Med Biol Soc* **2021**, *2021*, 7519-7525. DOI: 10.1109/EMBC46164.2021.9629539

(3) Clemente, F.; D'Alonzo, M.; Controzzi, M.; Edin, B. B.; Cipriani, C. Non-Invasive, Temporally Discrete Feedback of Object Contact and Release Improves Grasp Control of Closed-Loop Myoelectric Transradial Prostheses. *Ieee T Neur Sys Reh* **2016**, *24* (12), 1314-1322. DOI: 10.1109/Tnsre.2015.2500586

(4) Semere, A.; Payan, Y.; Cannard, F.; Diot, B.; Vuillerme, N. Using Sensory Substitution of Median Sensory Deficits in the Traumatized Hand to Develop an Innovative Home-Based Hand Rehabilitation System. *Inclusive Smart Cities and e-Health* **2015**, *9102*, 53-63. DOI: 10.1007/978-3-319-19312-0_5

(5) Hafidh, B.; Al Osman, H.; Alowaidi, M.; El-Saddik, A.; Liu, X. P. P. F-Glove: A Glove with Force-Audio Sensory Substitution System for Diabetic Patients. *2013 Ieee International Symposium on Haptic Audio-Visual Environments and Games (Have 2013)* **2013**, 34-38.

(6) Mendes, R. M.; Mazzer, N.; Zancheta, S.; Cavalcante, J. M. S. Reeducation of Sensibility of the Hand: Development of a Sensory Glove Model. *Acta Ortop Bras* **2011**, *19* (5), 289-292. DOI: Doi 10.1590/S1413-78522011000500005

(7) Trung, T. Q.; Le, H. S.; Dang, T. M. L.; Ju, S.; Park, S. Y.; Lee, N. E. Freestanding, Fiber-Based, Wearable Temperature Sensor with Tunable Thermal Index for Healthcare Monitoring. *Adv Healthc Mater* **2018**, *7* (12). DOI: 10.1002/adhm.201800074

(8) Yu, M. X.; Yu, G. B.; Dai, B. Graphene Fiber-Based Strain-Insensitive Wearable Temperature Sensor. *Ieee Sensor Lett* **2020**, *4* (10). DOI:10.1109/Lsens.2020.3026671

(9) Yu, Y. Y.; Peng, S. H.; Blanloeuil, P.; Wu, S. Y.; Wang, C. H. Wearable Temperature Sensors with Enhanced Sensitivity by Engineering Microcrack Morphology in PEDOT:PSS-PDMS Sensors. *Acs Appl Mater Inter* **2020**, *12* (32), 36578-36588. DOI: 10.1021/acsami.0c07649

(10) Kuzubasoglu, B. A.; Sayar, E.; Bahadir, S. K. Inkjet-Printed CNT/PEDOT:PSS Temperature Sensor on a Textile Substrate for Wearable Intelligent Systems. *Ieee Sens J* **2021**, *21* (12), 13090-13097. DOI: 10.1109/Jsen.2021.3070073

(11) Li, F.; Xue, H.; Lin, X. Z.; Zhao, H. R.; Zhang, T. Wearable Temperature Sensor with High Resolution for Skin Temperature Monitoring. *Acs Appl Mater Inter* **2022**, *14* (38), 43844-43852. DOI: 10.1021/acsami.2c15687

(12) Thiyagarajan, K.; Rajini, G. K.; Maji, D. Cost-Effective, Disposable, Flexible, and Printable MWCNT-Based Wearable Sensor for Human Body Temperature Monitoring. *Ieee Sens J* **2022**, *22* (17), 16756-16763. DOI: 10.1109/Jsen.2021.3088466

(13) Zhang, Z.; Li, Q. C.; Xu, L. Z.; Tian, W. H.; Li, Z. P. High-Performance Flexible Temperature Sensors Based on Laser-Irradiated Ag-MWCNTs/PEDOT:PSS. *Acs Appl Mater Inter* **2024**, *16* (5), 6078-6087. DOI: 10.1021/acsami.3c15734

(14) Heckmann, U.; Bandorf, R.; Gerdes, H.; Lübke, M.; Schnabel, S.; Bräuer, G. New materials for sputtered strain gauges. *Procedia Chem* **2009**, *1* (1), 64-67. DOI: 10.1016/j.proche.2009.07.016

(15) Jeong, Y. R.; Park, H.; Jin, S. W.; Hong, S. Y.; Lee, S. S.; Ha, J. S. Highly Stretchable and Sensitive Strain Sensors Using Fragmentized Graphene Foam. *Adv Funct Mater* **2015**, *25* (27), 4228-4236. DOI: 10.1002/adfm.201501000

(16) Zhang, M. C.; Wang, C. Y.; Wang, Q.; Jian, M. Q.; Zhang, Y. Y. Sheath-Core Graphite/Silk Fiber Made by Dry-Meyer-Rod-Coating for Wearable Strain Sensors. *ACS Appl. Mater. Inter.* **2016**, *8* (32), 20894-20899.
(17) Wang, N.; Xu, Z. Y.; Zhan, P. F.; Dai, K.; Zheng, G. Q.; Liu, C. T.; Shen, C. Y. A Tunable Strain Sensor Based on a Carbon Nanotubes/Electrospun Polyamide 6 Conductive Nanofibrous Network Embedded into Poly(vinyl alcohol) With Self-Diagnosis Capabilities. *J. Mater. Chem. C* **2017**, *5* (18), 4408-4418.
(18) Zheng, Y. J.; Li, Y. L.; Li, Z. Y.; Wang, Y. L.; Dai, K.; Zheng, G. Q.; Liu, C. T.; Shen, C. Y. The Effect of Filler Dimensionality on the Electromechanical Performance of Polydimethylsiloxane based Conductive Nanocomposites for Flexible Strain Sensors. *Compos. Sci. Technol.* **2017**, *139*, 64-73.
(19) Zhang, W. Y.; Liu, Q.; Chen, P. Flexible Strain Sensor Based on Carbon Black/Silver Nanoparticles Composite for Human Motion Detection. *Materials* **2018**, *11* (10).
(20) Wang, Y. L.; Jia, Y. Y.; Zhou, Y. J.; Wang, Y.; Zheng, G. Q.; Dai, K.; Liu, C. T.; Shen, C. Y. Ultra-Stretchable, Sensitive and Durable Strain Sensors Based on Polydopamine Encapsulated Carbon Nanotubes/Elastic Bands. *J. Mater. Chem. C* **2018**, *6* (30), 8160-8170.
(21) Wu, Y. C.; Karakurt, I.; Beker, L.; Kubota, Y.; Xu, R. X.; Ho, K. Y.; Zhao, S. L.; Zhong, J. W.; Zhang, M.; Wang, X. H.; et al. Piezoresistive Stretchable Strain Sensors with Human Machine Interface Demonstrations. *Sensor. Actuat. A: Phys.* **2018**, *279*, 46-52.
(22) Yan, T.; Zhou, H.; Niu, H. T.; Shao, H.; Wang, H. X.; Pan, Z. J.; Lin, T. Highly Sensitive Detection of Subtle Movement Using a Flexible Strain Sensor from Helically Wrapped Carbon Yarns. *J. Mater. Chem. C* **2019**, *7* (32), 10049-10058.
(23) Li, H.; Zhang, J. J.; Chen, J.; Luo, Z. B.; Zhang, J. Y.; Alhandarish, Y.; Liu, Q. H.; Tang, W.; Wang, L. A Supersensitive, Multidimensional Flexible Strain Gauge Sensor Based on Ag/PDMS for Human Activities Monitoring. *Sci. Rep.* **2020**, *10* (1).
(24) Mechael, S. S.; Wu, Y. Y.; Chen, Y. T.; Carmichael, T. B. Ready-to-Wear Strain Sensing Gloves for Human Motion Sensing. *Iscience* **2021**, *24* (6).
(25) Si, Y. Y.; Chen, S. J.; Li, M.; Li, S. Y.; Pei, Y. S.; Guo, X. J. Flexible Strain Sensors for Wearable Hand Gesture Recognition: From Devices to Systems. *Adv. Intell. Syst.* **2022**, *4* (2).
(26) Bozali, B.; Ghodrat, S.; Jansen, K. M. B. Design of Wearable Finger Sensors for Rehabilitation Applications. *Micromachines* **2023**, *14* (4).
(27) Cheng, H. Y.; Zhang, X.; Zhang, T.; Chen, Y. X.; Yu, D.; Wang, W. Durable TPU/PDA/MXene Fiber-Based Strain Sensors with High Strain Range and Sensitivity. *Chemistryselect* **2024**, *9* (31).
(28) Cai, L.; Song, L.; Luan, P. S.; Zhang, Q.; Zhang, N.; Gao, Q. Q.; Zhao, D.; Zhang, X.; Tu, M.; Yang, F.; et al. Super-Stretchable, Transparent Carbon Nanotube-Based Capacitive Strain Sensors for Human Motion Detection. *Sci. Rep.* **2013**, *3*.
(29) Wang, X. Y.; Deng, Y.; Jiang, P.; Chen, X. R.; Yu, H. Y. Low-Hysteresis, Pressure-Insensitive, and Transparent Capacitive Strain Sensor for Human Activity Monitoring. *Microsyst. Nanoeng.* **2022**, *8* (1).
(30) Duan, S. S.; Wang, J. Y.; Lin, Y.; Hong, J. L.; Lin, Y. C.; Xia, Y. R.; Li, Y. H.; Zhu, D.; Lei, W.; Su, W. M.; et al. Highly Durable Machine-Learned Waterproof Electronic Glove Based on Low-Cost Thermal Transfer Printing for Amphibious Wearable Applications. *Nano Res.* **2023**, *16* (4), 5480-5489.
(31) Au, C. Y.; Leow, S. Y.; Yi, C. X.; Ang, D.; Yeo, J. C.; Koh, M. J. A.; Bhagat, A. A. S. A Sensorised Glove to Detect Scratching for Patients with Atopic Dermatitis. *Sensors* **2023**, *23* (24).

(32) Duan, S. S.; Lin, Y. C.; Zhang, C. Y.; Li, Y. H.; Zhu, D.; Wu, J.; Lei, W. Machine-Learned, Waterproof MXene Fiber-Based Glove Platform for Underwater Interactivities. *Nano Energy* **2022**, *91*.
(33) Deng, L. N.; Shen, Y.; Hong, Y.; Dong, Y. L.; He, X.; Yuan, Y.; Li, Z.; Ding, H. Sen-Glove: A Lightweight Wearable Glove for Hand Assistance with Soft Joint Sensing. *2022 IEEE Int. Conf. Robot. Autom.* **2022**, 5170-5175.
(34) Oh, J.; Kim, S.; Lee, S.; Jeong, S.; Ko, S. H.; Bae, J. A Liquid Metal Based Multimodal Sensor and Haptic Feedback Device for Thermal and Tactile Sensation Generation in Virtual Reality. *Adv. Funct. Mater.* **2021**, *31* (39).
(35) Chen, X. S.; Gong, L.; Wei, L.; Yeh, S. C.; Xu, L. D.; Zheng, L. R.; Zou, Z. A Wearable Hand Rehabilitation System With Soft Gloves. *IEEE T. Ind. Inform.* **2021**, *17* (2), 943-952.
(36) Hosseini, M.; Sengül, A.; Pane, Y.; De Schutter, J.; Bruyninckx, H. ExoTen-Glove: A Force-Feedback Haptic Glove Based on Twisted String Actuation System. *IEEE Roman* **2018**, 320-327.
(37) Choi, Y.; Yoo, K.; Kang, S. J.; Seo, B.; Kim, S. K. Development of a Low-Cost Wearable Sensing Glove with Multiple Inertial Sensors and a Light and Fast Orientation Estimation Algorithm. *J. Supercomput.* **2018**, *74* (8), 3653-3653.
(38) Ryu, D.; Moon, K. W.; Nam, H.; Lee, Y.; Chun, C.; Kang, S.; Song, J. B. Micro Hydraulic System Using Slim Artificial Muscles for a Wearable Haptic Glove. *2008 IEEE/RSJ Int. Conf. on Robots Intelligent Sys.* **2008**, 3028.
